# The lasting impact of formation cycling on the Li-ion kinetics between SEI and the Li-metal anode and its correlation with efficiency

**DOI:** 10.1126/sciadv.adj8889

**Published:** 2024-01-17

**Authors:** Shengnan Zhang, Yuhang Li, Lars J. Bannenberg, Ming Liu, Swapna Ganapathy, Marnix Wagemaker

**Affiliations:** ^1^ Section Storage of Electrochemical Energy, Radiation Science and Technology, Faculty of Applied Sciences, Delft University of Technology, Mekelweg 15, 2629 JB Delft, Netherlands.; ^2^ Shenzhen Key Laboratory of Power Battery Safety and Shenzhen Geim Graphene Center, Tsinghua Shenzhen International Graduate School, Tsinghua University, Guangdong 518055, China.

## Abstract

Formation cycling is a critical process aimed at improving the performance of lithium ion (Li-ion) batteries during subsequent use. Achieving highly reversible Li-metal anodes, which would boost battery energy density, is a formidable challenge. Here, formation cycling and its impact on the subsequent cycling are largely unexplored. Through solid-state nuclear magnetic resonance (ssNMR) spectroscopy experiments, we reveal the critical role of the Li-ion diffusion dynamics between the electrodeposited Li-metal (ED-Li) and the as-formed solid electrolyte interphase (SEI). The most stable cycling performance is realized after formation cycling at a relatively high current density, causing an optimum in Li-ion diffusion over the Li-metal-SEI interface. We can relate this to a specific balance in the SEI chemistry, explaining the lasting impact of formation cycling. Thereby, this work highlights the importance and opportunities of regulating initial electrochemical conditions for improving the stability and life cycle of lithium metal batteries.

## Teaser

Li-ion diffusion dynamics hold a critical role between Li-metal anode and SEI, which has a lasting impact of formation cycling.

## INTRODUCTION

Following the introduction of the metallic lithium (Li) electrode by Whittingham in the 1970s, it remains to date a topic of great research interest. This is because Li-metal has more than 10 times the specific capacity (3860 mAh g^−1^) of current commercial carbonaceous anodes and the lowest potential (−3.040 V versus the standard hydrogen electrode) (*1*, *2*). However, its commercialization remains challenging as severe problems persist, such as the inherent high reactivity with the electrolyte and the nonuniform deposition upon plating/stripping, causing poor reversibility and thus a short life cycle (*3*, *4*). In general, Li-metal reduces the electrolyte on contact forming an interfacial layer, referred to as the solid electrolyte interface (SEI), with a thickness in the order of nanometers. During SEI formation, the electrolyte is reduced, which passivates a fraction of the Li inventory of the cell. Ideally, the SEI forms a mechanically stable interphase that prevents further electrolyte reduction while not posing a barrier to lithium ion (Li-ion) transport (*5*–*7*). However, the low surface energy of Li-metal easily leads to the formation of high surface area mossy and dendritic Li-metal morphologies. Each time, this leads to fracturing of the SEI that exposes fresh Li-metal to the electrolyte. This induces further SEI formation and thus irreversible capacity loss (*7*, *8*). In addition, mossy and dendritic structures lead to electrical disconnected Li-metal upon repeated plating and stripping, inducing further Li losses. This self-amplifying process, the extent of which depends on the choice of electrolyte, current collector, and cycling conditions, is responsible for poor reversibility of the Li-metal electrode. This is expressed by the Coulombic efficiency (CE), i.e., the ratio of the electrons passed on discharge to those passed on charge (*9*). Maintaining a uniform and dense Li-metal morphology, preventing uncontrolled SEI formation and contact losses, is considered to be a prerequisite for achieving a high CE and life cycle for lithium metal batteries (LMBs). 

It has been shown that the electrokinetics at the surface of Li-metal plays a central role in the evolution of both its morphology and the SEI formation (*4*, *10*, *11*). Whether the electroplating process occurs at a buried Li-SEI interface or at a fresh Li-electrolyte interface is closely related to the applied current density (*8*, *12*–*14*). When electroplating occurs at a buried Li-SEI interface, the SEI regulates the Li-ion exchange between the electrolyte and Li-metal, where higher exchange rates have been associated with high CE, typically increasing during cycling for electrolytes that provide high CE (*15*, *16*). For electroplating at the fresh Li-electrolyte interface, the strength of Li-ion solvation in different electrolytes has been proposed to influence a preference for dendritic or uniform morphologies (*12*, *17*). In principle, Li-metal growth is sensitive to a range of factors, including electrolyte chemistry, current density, formation cycles, temperature, and internal pressure, among others (*18*–*21*). Understanding the role of each of these is essential in realizing an optimal Li-metal anode performance.

The impact of the formation cycling conditions on the initial SEI morphology and its properties is one such factor that can be anticipated to have a long lasting impact on subsequent cycling (*14*, *22*–*24*). This phenomenon has been explored using electrochemical approaches such as pulse-current or cyclic voltammetry premodulation, which have tentatively probed the influence of initial current densities used on the SEI and the Li morphologies (*25*–*27*). For instance, the pulse current protocol improves the cycling performance through higher nucleation densities during initial Li deposition and homogenous SEI film formation (*27*). Previous work observed that during initial medium-high cycling rates, more compact Li microstructures formed, suggesting that this results in a templated SEI that defines the subsequent Li-metal morphology (*28*). It was also found that for a weakly solvating, high-performance electrolyte, small formation current densities (0.5 mA cm^−2^) lead to a more porous SEI, where it was postulated that the Li-metal, formed during subsequent cycles, nucleated within the SEI, suppressing electrolyte decomposition and explaining the observed improved CE (*14*). Therefore, in a certain current density range, the SEI formed at higher rates is more compact with a more uniform Li-metal morphology, while at lower rates, the SEI is more porous and with less dense Li-metal morphology.

These findings indicate that the initially formed SEI has a large impact on the subsequent cycling performance of Li-metal anodes (*8*, *14*, *17*, *28*, *29*). Nevertheless, comprehensive understanding of the underlying mechanisms is lacking, especially because of the diversity of electrolyte chemistries that have been studied (*12*, *17*, *30*). A central factor in this is the influence of the properties of the initially formed SEI on the Li-metal morphology and Li-ion charge-transfer kinetics, and how these influence subsequent long-term cycling. Several in-depth studies have been performed to evaluate the SEI morphology and composition, typically using cryo–transmission electron microscopy (TEM) in combination with advanced analysis of electrochemical impedance and voltammetry measurements (*22*, *26*, *30*–*34*). Although the role of the local Li-ion kinetics both in the SEI and between the SEI, Li-metal, and the electrolyte is believed to play an important role, it remains ambiguous to date. This is mainly because it is very challenging to measure the local Li-ion kinetics of the SEI experimentally. Isotope selective solid-state nuclear magnetic resonance (ssNMR) offers opportunities to probe the Li-ion diffusivity between distinguishable Li environments. Recently, one-dimensional (1D) and 2D exchange ssNMR have been used to identify the role of the LiNO_3_ additive to an ether-based electrolyte and qualitatively probe the growth of Li dendrites (*35*, *36*). In addition, the SEI chemistry can be characterized using multinuclear magic angle spinning (MAS) ssNMR (ex situ) in conjunction with x-ray photoelectron spectroscopy (XPS), while operando NMR allows quantification of the Li microstructure and the SEI capacity (*37*, *38*).

Here, we study the influence of the formation current density on the Li-ion diffusion kinetics between the SEI and the electrodeposited Li-metal (ED-Li), and how altering the electrochemical preconditioning and the formation cycles affect subsequent cycling. Different formation cycle current densities ranging from 0.2 to 5 mA cm^−2^ are studied in terms of CE and cycling stability. The Li-ion kinetics and SEI properties are evaluated by (i) variable temperature (VT) exchange ssNMR to quantify the kinetics of Li-ion migration between the two solid phases: ED-Li and SEI; (ii) cross-polarization (CP) ssNMR experiments to assign the chemical components in the SEI; and (iii) operando NMR, where the influence of electrochemical preconditioning on dead Li formation has been noninvasively and directly monitored. Complemented by scanning electron microscopy (SEM) and XPS, the equilibrium Li-ion flux between ED-Li and SEI is correlated with the SEI composition for different current density formation cycles. A relatively high formation current density (2 mA cm^−2^) facilitates the local Li-ion transport, which is correlated by a specific balance in the inorganic SEI species. This is found to be the origin for the improved reversibility and cycling stability, increasing the CE and minimizing the formation of dead Li-metal. In this manner, the present research uncovers the Li-metal ⇋ SEI kinetics during formation cycling and how this affects subsequent cycling performance, providing practical guidelines for formation cycling protocols to be used in LMBs.

## RESULTS

### The impact of electrochemical preconditioning on the LMBs performance

To identify the effect of formation cycling, Li||Cu half cells were assembled using a commercial carbonate-based 1 M LiPF_6_ (lithium hexafluorophosphate) in ethylene carbonate/dimethyl carbonate [EC/DMC; 1:1 (w/w)] electrolyte. The cells were subjected to different formation conditions by decreasing the current density used during the five formation cycles from 5 to 0.2 mA cm^−2^ guided by previous research (*22*, *28*). In addition, formation cycles at a current density of 0.5 mA cm^−2^ were performed as a blank reference (control group). Following the five formation cycles, all the cells were cycled for more than 400 hours at 0.5 mA cm^−2^ to an areal plating capacity of 1 mAh cm^−2^ for each cycle.

**Fig. 1.**
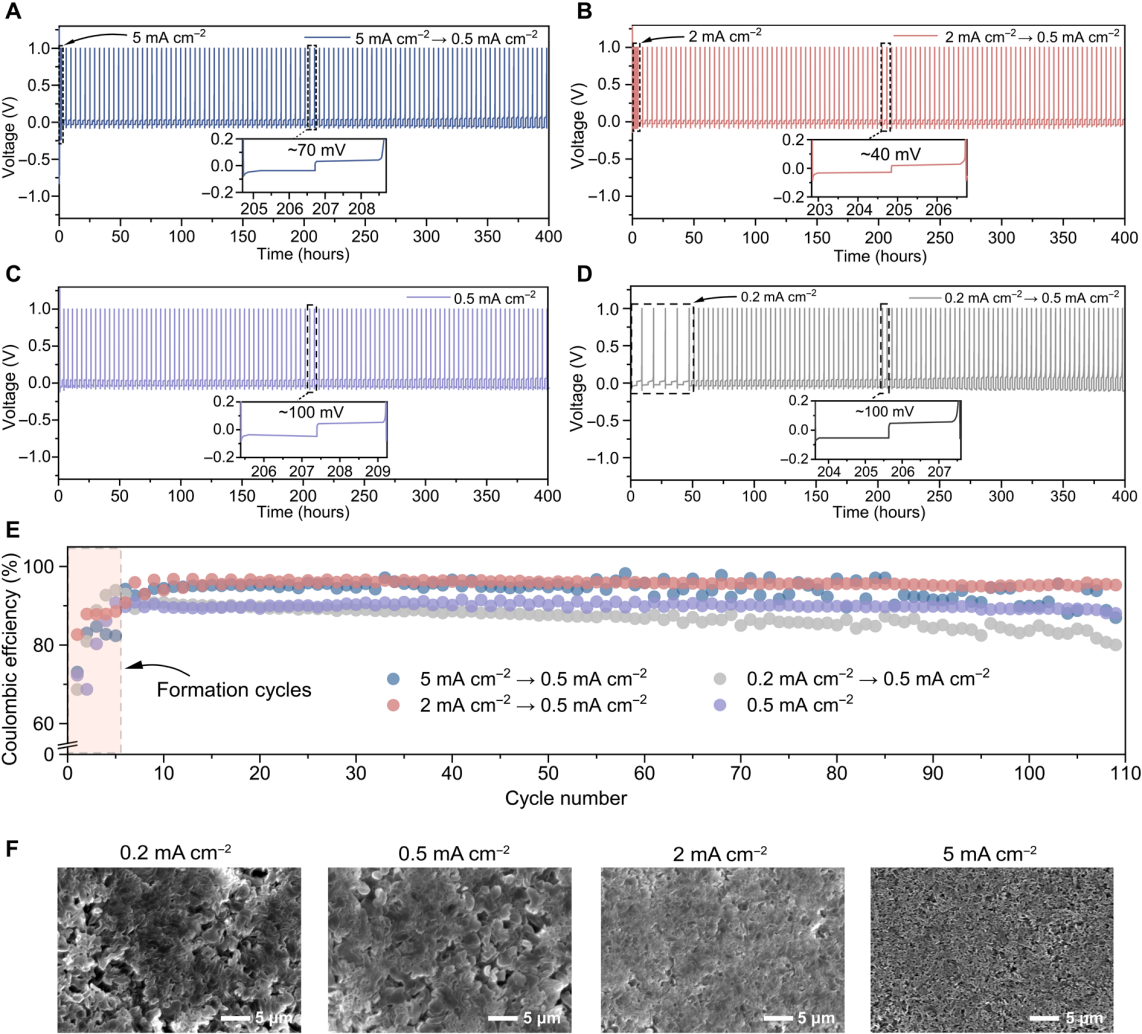
Electrochemical characterization of the LMBs with different formation current densities. Galvanostatic cycling performance and polarization profiles of the Li||Cu cells cycled at: (**A**) 5 to 0.5 mA cm^−2^, (**B**) 2 to 0.5 mA cm^−2^, (**C**) 0.5 mA cm^−2^, and (**D**) 0.2 to 0.5 mA cm^−2^. Five formation cycles were applied in advance, Li was electrodeposited to a capacity of 1 mAh cm^−2^ for each plating step, and inserts are the enlarged view of the voltage profiles at 202 to 209 hours, indicating the overpotential values. (**E**) Comparison of the corresponding CE for the Li||Cu cells cycled at the current densities given in (A) to (D). (**F**) SEM images showing the morphology of the Li-metal plated on Cu in Li||Cu half cells after a single plating to a total capacity of 1 mAh cm^−2^ at current densities of 0.2, 0.5, 2, and 5 mA cm^−2^, respectively.

As seen in Fig. 1, the electrochemical cycling highlights that the formation current density has a pronounced impact on the subsequent cycles. Comparing the four different formation protocols, a formation current density of 0.2 mA cm^−2^ (Fig. 1D) initially displays a gradual increase in overpotential from the start of the working cycles (cycles after formation), which is notably accelerated after 200 hours of cycling, resulting in an overpotential of about 100 mV (Fig. 1D, insert), similar to that continuously cycled at 0.5 mA cm^−2^ (Fig. 1C, insert). For formation cycles at a relatively high current density of 2 mA cm^−2^, a smaller increase in overpotential of ~40 mV is observed after ~205 hours (Fig. 1B, insert). The cell with a formation current density of 5 mA cm^−2^ results again in a higher overpotential of ~70 mV at ~206.5 hours (Fig. 1A, insert), suggesting that there is an optimal formation current density around 2 mA cm^−2^ at which the overpotential is minimal. The magnitude of the overpotential plateau reflects the internal resistance, which could be a reflection of the effective conductivity between the electrolyte and Li-metal, thus related to the SEI properties. This trend agrees with the impedance results reported by Xu *et al.* (*22*), where the minimum resistance of the deposited Li was also found to be at 2 mA cm^−2^. A similar trend is also observed in the comparison of the CE. The cell cycled at a formation current density of 2 mA cm^−2^ results in the highest CE during the subsequent cycling (0.5 mA cm^−2^), stabilizing around ~96% for more than 100 cycles, while the cell cycled from 0.2 to 0.5 mA cm^−2^ shows an obvious fading of the CE to around 80% (Fig. 1E). The reference cell, cycled constantly at 0.5 mA cm^−2^, maintains a CE of around 88%. A formation cycling density of 5 mA cm^−2^ results in fluctuations and a decreasing CE.

Post-mortem SEM images (Fig. 1F) are taken to visualize the differences in Li-metal morphology caused by using different initial current densities. For lithium plated on a Cu foil at 0.2 mA cm^−2^, porous Li deposits are observed, sparsely distributed over the Cu foil surface with tangible Li whiskers, forming spongy Li domains with an average particle size of ~5 μm. While the morphology of Li-metal plated at 0.5 mA cm^−2^ is similar to that at 0.2 mA cm^−2^, it shows a denser column-like morphology having smaller Li-metal deposit sizes. In contrast, the Li-metal morphology formed at 2 mA cm^−2^ is much denser and compact with a more uniform microstructure and smaller Li deposits of around 1 μm. For the ED-Li deposited at 5 mA cm^−2^, the Li-metal morphology is more porous again. The morphology of the ED-Li has been further studied by TEM as shown in fig. S1. The ED-Li has a different size distribution where 2 and 5 mA cm^−2^ show smaller size than the other two (fig. S1, A to D), and the observed SEI thicknesses are ~28, ~23, ~14, and ~36 nm for the deposition current densities of 0.2, 0.5, 2, and 5 mA cm^−2^, respectively (fig. S1, E to H). The thinner SEI formed at 2 mA cm^−2^ is potentially favorable for faster Li-ion transport by reducing the ion conduction path lengths and consequently the ionic resistance.

This trend is consistent with literature: The Li nucleation size decreases with increasing current density, and the distribution of the deposited Li particles changes from densely packed to overlapping stacked multilayers (*22*, *23*, *39*). However, the performance of LMBs can vary greatly with the electrolyte composition. To see the impact of the formation current on different electrolyte formulations, we compared three extra representative electrolytes by applying the same electrochemical test protocols: a modified carbonate-based electrolyte with fluoroethylene carbonate (FEC) and vinylene carbonate (VC) additives {1 M LiPF_6_ in EC/DMC [1:1 (w/w)] with 10 wt % FEC and 1 wt % VC}, an ether-based electrolyte {1 M lithium bis(trifluoromethane sulfonyl)imide (LiTFSI) in 1,2-dimethoxyethane/1,3-dioxolane (DME/DOL) [1:1 (w/w)]} and a modified ether-based electrolyte with a lithium nitrate (LiNO_3_) additive {1 M LiTFSI in DME/DOL [1:1 (w/w)] with 5 wt % LiNO_3_}. For the Li||Cu cells cycled with the modified carbonate-based electrolyte (fig. S2), similar trends have been found that using 2 and 5 mA cm^−2^ as formation current density improved the cycling stability and efficiency of the subsequent cycles. In the standard ether-based electrolyte (fig. S3), 1 M LiTFSI in DOL/DME, a formation current density of 2 mA cm^−2^ does not show as evident an improvement; however, it does result in better stability and lower polarization compared to other cycling protocols. When using the ether-based electrolyte with the functional additive LiNO_3_ (fig. S4), the impact of the formation current density is similar to what we have observed for the ether-based electrolyte without additive shown in fig. S3, but the cells show smaller polarization and slightly higher CE, which can be attributed to the improved SEI by LiNO_3_ reduction products (*40*). On the basis of the cycling performance of these electrolytes, it is reasonable to conclude that the current density applied during the formation cycles is instructive for optimizing the LMBs performance.

Nevertheless, the relationship between the initially formed SEI properties and the long-term battery performance is not established. The low overpotential observed on long-term cycling, after formation cycling at 2 mA cm^−2^, suggests that facile Li-ion transport can be attributed to the presence of a highly conductive and stable SEI. As the SEI is generated during the formation cycles, it is also plausible that it forms a scaffold within which the Li-metal will be plated during subsequent cycles, and thus the morphology and properties of this SEI scaffold will play a large role in the reversibility (*22*). The correlation between the formation current density and SEI conductivity and structure is explored in greater depth in the subsequent sections.

### Li-ion diffusion between the ED-Li and the SEI

**Fig. 2.**
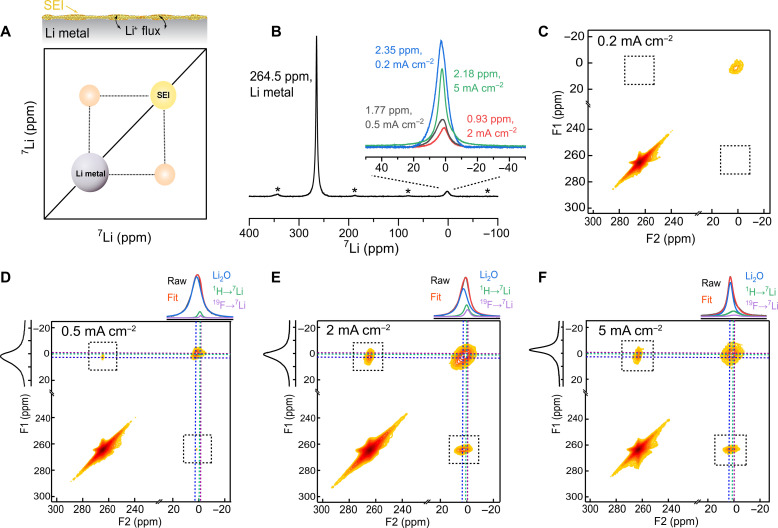
^7^Li-^7^Li 2D exchange ssNMR experiments measuring Li-ion transport between the initially plated ED-Li and the as-formed SEI. (**A**) Schematic showing the ^7^Li-^7^Li 2D Li^+^ exchange between the ED-Li (gray) and the SEI (yellow). (**B**) 1D ^7^Li MAS spectrum of the ED-Li and SEI generated at 5 mA cm^−2^ (green), 2 mA cm^−2^ (red), 0.5 mA cm^−2^ (gray), and 0.2 mA cm^−2^ (blue), respectively (insert). The black spectrum corresponds to the Li-metal peak plated at 2 mA cm^−2^ (see the other three in fig. S6). Asterisks denote spinning sidebands. (**C** to **F**) 2D ^7^Li-^7^Li exchange spectroscopy (2D-EXSY) of ED-Li and its corresponding SEI formed at: (C) 0.2 mA cm^−2^, (D) 0.5 mA cm^−2^, (E) 2 mA cm^−2^, and (F) 5 mA cm^−2^ at *T*_mix_ = 150 ms and room temperature (298 K). The projections in F2 and F1 displayed in (D) to (F) correspond to the 1D slices of the SEI and the exchange peaks, aiming at assigning the components that dominate the exchange. The cross-peak at the off-diagonal positions in the dashed boxes represent the diffusion of Li-ions between the ED-Li and the as-formed SEI.

The direct detection of the spontaneous Li-ion diffusion between the ED-Li and the SEI is feasible by 2D exchange spectroscopy (2D-EXSY) ssNMR experiments under MAS conditions (*41*–*46*). All the samples were measured ex situ, and therefore, the Li-ion exchange represents the equilibrium exchange current density between Li-metal and SEI, which is representative of the Li-ion kinetics between these two phases under equilibrium conditions. Therefore, this includes the charge transfer kinetics at equilibrium (the exchange current density), most likely also Li-ion diffusion through the SEI, but does not include the (de)solvation kinetics between the SEI and liquid electrolyte. Exchange between the detected resonances within the NMR time scale gives rise to off-diagonal (cross) peaks in the 2D contour plots (Fig. 2A). On incrementing the mixing time *T*_mix_, it allows more time for the Li-ions to migrate from one environment to another, resulting either in an increase in cross-peak intensity or the appearance of additional cross peaks between less mobile or spatially distant Li-containing environments (see the pulse sequence in fig. S5). Similar observations can be made when performing the experiment at elevated temperatures as Li-ions become more mobile, resulting in faster dynamics (exchange). Therefore, the 2D-EXSY technique allows us to quantify the Li-ion exchange and thus the charge transfer kinetics between the ED-Li and the as-formed SEI phases, similar to what has been showcased for ether-based electrolytes (*36*).

To discriminate the different Li-containing species present in the ED-Li and its SEI after directly plating Li on a Cu substrate at 0.2, 0.5, 2, and 5 mA cm^−2^, one-pulse ^7^Li MAS ssNMR spectra were measured, shown in Fig. 2B. The black spectrum corresponds to 2 mA cm^−2^ (the spectra corresponding to Li-metal plated at 0.2, 0.5, and 5 mA cm^−2^ are provided in the fig. S6). Two distinguishable resonances can be observed in the spectrum corresponding to two main kinds of Li environments, one representing metallic Li [~264.5 parts per million (ppm)] and the other representing Li species in the SEI generated (Fig. 2B, insert). It is worth noting that the chemical shift of the main diamagnetic SEI species depends on the formation current density. It is observed at 2.18, 0.93, 1.77, and 2.35 ppm for the plating current densities of 5, 2, 0.5, and 0.2 mA cm^−2^, respectively. The differences originate from the dominant Li-containing chemical species inside the SEI, which we will discuss in detail in the following sections.

From the 2D exchange spectra measured at *T*_mix_ = 150 ms at room temperature, there are no cross-peaks observed between Li-metal and SEI for the sample electrodeposited at a small current density of 0.2 mA cm^−2^ (ED-0.2; Fig. 2C). When deposited at 0.5 mA cm^−2^ (ED-0.5; Fig. 2D) and 1 mA cm^−2^ (ED-1; fig. S7B), cross-peaks emerge (indicated by the dashed black box), reflecting equilibrium Li-ion exchange diffusion between Li-metal and the SEI. At the same mixing time, more intense cross-peaks are observed for 2 and 5 mA cm^−2^ (ED-2 and ED-5) (Fig. 2, E and F), indicating more equilibrium exchange between SEI and Li-metal deposited at higher current densities. Results obtained with electrochemical impedance spectroscopy (EIS) after five formation cycles show the same trend (fig. S11B), which we have discussed in detail in the next section. The resistance ascribed to the SEI decreases with increasing formation current density (2 mA cm^−2^ < 0.5 mA cm^−2^ < 0.2 mA cm^−2^), which is in agreement with the lack of/lower cross-peak intensities observed at 0.2 and 0.5 mA cm^−2^, respectively. When the measurements were performed at an elevated temperature (343 K), cross-peaks were also observed for the ED-0.2 sample (fig. S7A), which is a consequence of the thermally enhanced Li-ion exchange.

### Quantification of the Li-ion exchange

**Fig. 3.**
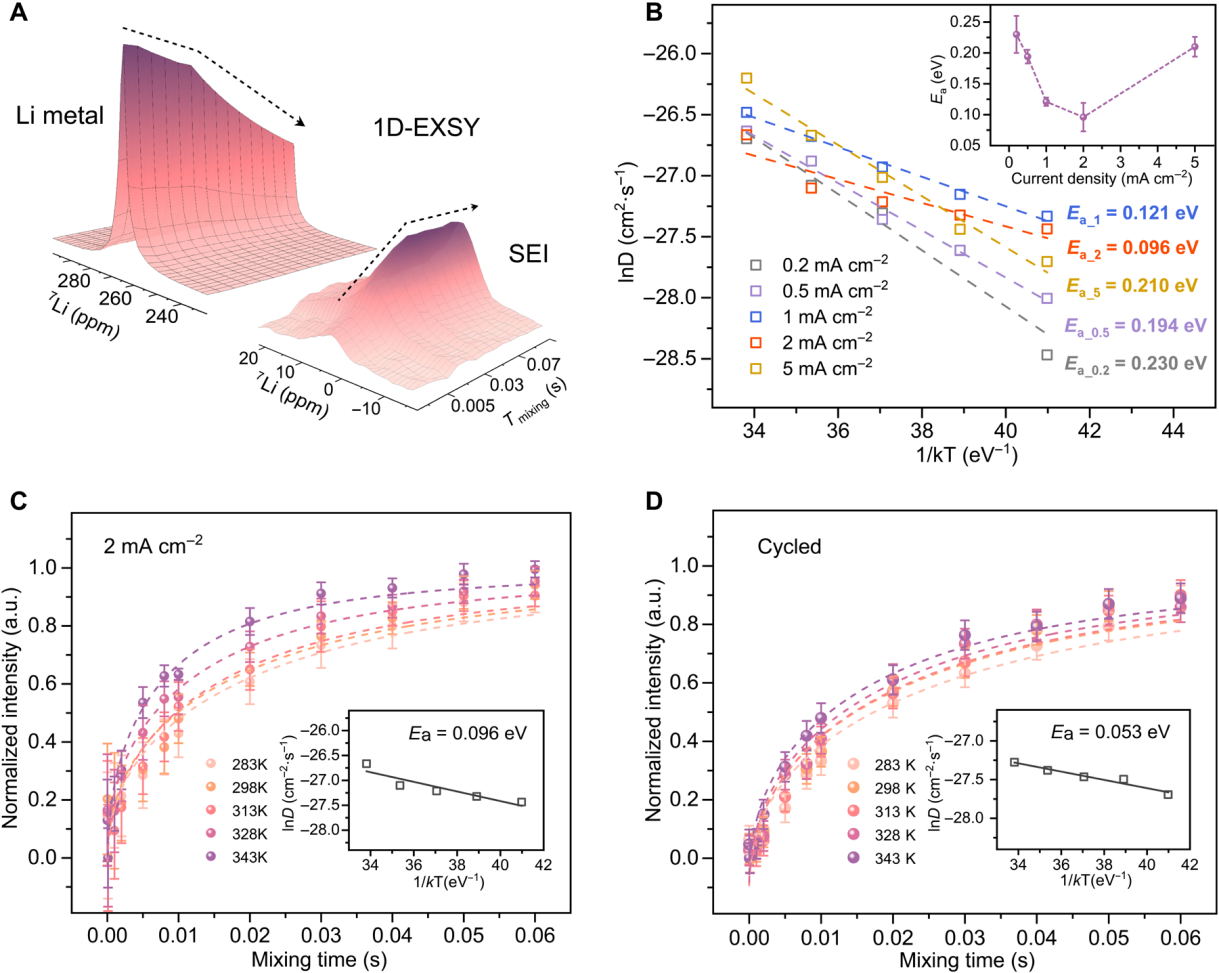
Quantification of Li-ion diffusion between ED-Li and SEI by ^7^Li 1D ssNMR exchange experiments. (**A**) Schematic illustration of the Li-ion transport in the 1D ^7^Li exchange spectroscopy measurements (1D-EXSY) by probing the gradual diminishing of ED-Li peak and the increase of SEI peak as a function of mixing time (*T*_mix_). (**B**) Dependence of the diffusion coefficient (*D*) obtained from fitting the 1D-EXSY data to a diffusion model has been described by us in detail elsewhere (*47*, *66*). 1D-EXSY of the initially plated ED-Li and the SEI plated at (**C**) 2 mA cm^−2^ and the cycled samples of (**D**) 2 to 0.5 mA cm^−2^. For the cycled sample, five formation cycles at 2 mA cm^−2^ are first used, followed by 10 cycles at 0.5 mA cm^−2^. One final deposition process at 0.5 mA cm^−2^ is adopted at the end of the cycles to keep the sample amount consistent. Error bars in (B) inset represent the SE of the fitting, and error bars in (C) and (D) represent the SD of the spectrum noise. a.u., arbitrary units.

Given that the exchange experiments only measure the absolute amount of exchanged Li, this will scale with the Li-metal-SEI interface area, which is difficult, if not impossible to assess. The largest amount of Li-ion exchange between SEI and Li-metal is observed after plating at 2 mA cm^−1^, thus for the most densely plated Li-metal morphologies (Fig. 1F), which is expected to have the smallest interface area with the SEI. This suggests that the Li-ion kinetics between SEI and Li-metal is much better after formation cycling at this current density (2 mA cm^−1^). To access the kinetics quantitatively, the activation energy (*E*_a_) for the Li-ion exchange diffusion between SEI and Li-metal is determined by exchange experiments performed at VTs. Since the diamagnetic Li-species in the SEI have a very long spin-lattice relaxation time (*T*_1_) and that of metallic Li is much shorter, 1D ^7^Li exchange spectroscopy (1D-EXSY) ssNMR experiments can be used, a more time efficient method to quantify the Li-ion diffusion compared to the 2D-EXSY measurements. In the 1D-EXSY experiments, the SEI resonance is initially selectively filtered out using a *T*_2_ filter (*44*, *47*), after which the magnetization transfer from the Li-metal back to the SEI is monitored as a function of mixing time *T*_mix_. As depicted in Fig. 3A, at very short mixing times *T*_mix_ = 0.00001 s, only a tiny resonance signal was observed for the SEI. Increasing the mixing time, the Li in the metal phase exchanges with the Li-ion species in the SEI phase, which is responsible for the intensity decrease of the Li-metal resonance and the increase of the SEI resonance. This is evidenced in fig. S8 where the 1D spectra corresponding to short and long mixing times are overlaid for the ED-2 sample.

The VT 1D-EXSY reveals evident differences in Li-ion exchange dynamics for the electrodeposited samples depending on the formation current density. The normalized intensities of the evolving ^7^Li SEI signal as a function of *T*_mix_ are shown in Fig. 3C and fig. S9. The Li-ion diffusion between the ED-Li and SEI was quantified by fitting the emerging SEI signal as a function of *T*_mix_ to a diffusion model derived from Fick’s law applied to describe the spontaneous equilibrium Li-ion exchange between two solid phases (see text S1) (*47*). The average self-diffusion coefficient (*D*_Li_) as a function of temperature obtained from fitting the data to this model pertains to the Li-ion transfer between the ED-Li and the SEI. An Arrhenius law was used to fit *D*_Li_ obtained for various temperatures from which the *E*_a_ for each case was determined (see Fig. 3B). The inset in Fig. 3B shows that the lowest energy barrier is observed for ED-2, 0.096 eV, and the highest barrier for ED-0.2, 0.23 eV. This large span of *E*_a_ values (more than a factor of 2) shows that Li-ion diffusion between the two solid-state phases (ED-Li and SEI) strongly depends on the current density during formation cycling. For a much higher formation current density of 5 mA cm^−2^, *E*_a_ increases again, leading to a minimum/optimum current density with respect to achieving a low *E*_a_ for Li-ion exchange between SEI and Li-metal. The trend of *E*_a_ versus formation current density correlates to the electrochemical performance shown in Fig. 1, where the CE is inversely related to the *E*_a_ for Li-ion exchange between the SEI and the ED-Li. In other words, more facile Li-ion kinetics between SEI and Li-metal correlates to a higher CE.

To evaluate if the charge transfer kinetics set by the formation current density remains upon subsequent cycling, additional ED-Li and SEI samples were measured with 1D-EXSY after five formation cycles at 2 mA cm^−2^ followed by 10 cycles at 0.5 mA cm^−2^ (see Fig. 3D). Compared to right after plating, the additional cycles reduce the *E*_a_ by a factor of 2 (0.053 eV). For comparison, the same experiments were performed for the lower formation current densities, five formation cycles at 0.2 or 0.5 mA cm^−2^, followed by 10 subsequent cycles at 0.5 mA cm^−2^, also resulting in a decrease in *E*_a_ from 0.23 to 0.159 eV and from 0.194 to 0.093 eV, respectively (fig. S10). We can thus conclude that the *E*_a_ for Li-ion exchange between SEI and Li-metal, set by the formation cycling, has a lasting impact during subsequent cycling. Moreover, subsequent cycling reduces *E*_a_; hence, the Li-ion kinetics improve, reflecting better ionic contact between SEI and Li-metal. EIS measurements, performed on the cells after 10 subsequent working cycles (fig. S11), show a similar trend with resistances decreasing even further after the working cycles, with the biggest decrease seen for cells with a formation cycle current density of 2 mA cm^−2^. On extended cycling, however, i.e., 50 and 100 working cycles, respectively, the cell resistance increases. This is also unsurprising as it is expected that more Li-metal reacts with the electrolyte during long cycling due to which the as-formed SEI inevitably experiences repetitive breakage/regeneration, resulting in the thickness, composition, and structure of the SEI to change accordingly (*4*, *5*, *48*). This indicates that the nature of these interfaces evolve during cycling, although the origin of this cannot be concluded based on these results.

### Composition of the SEI

**Fig. 4.**
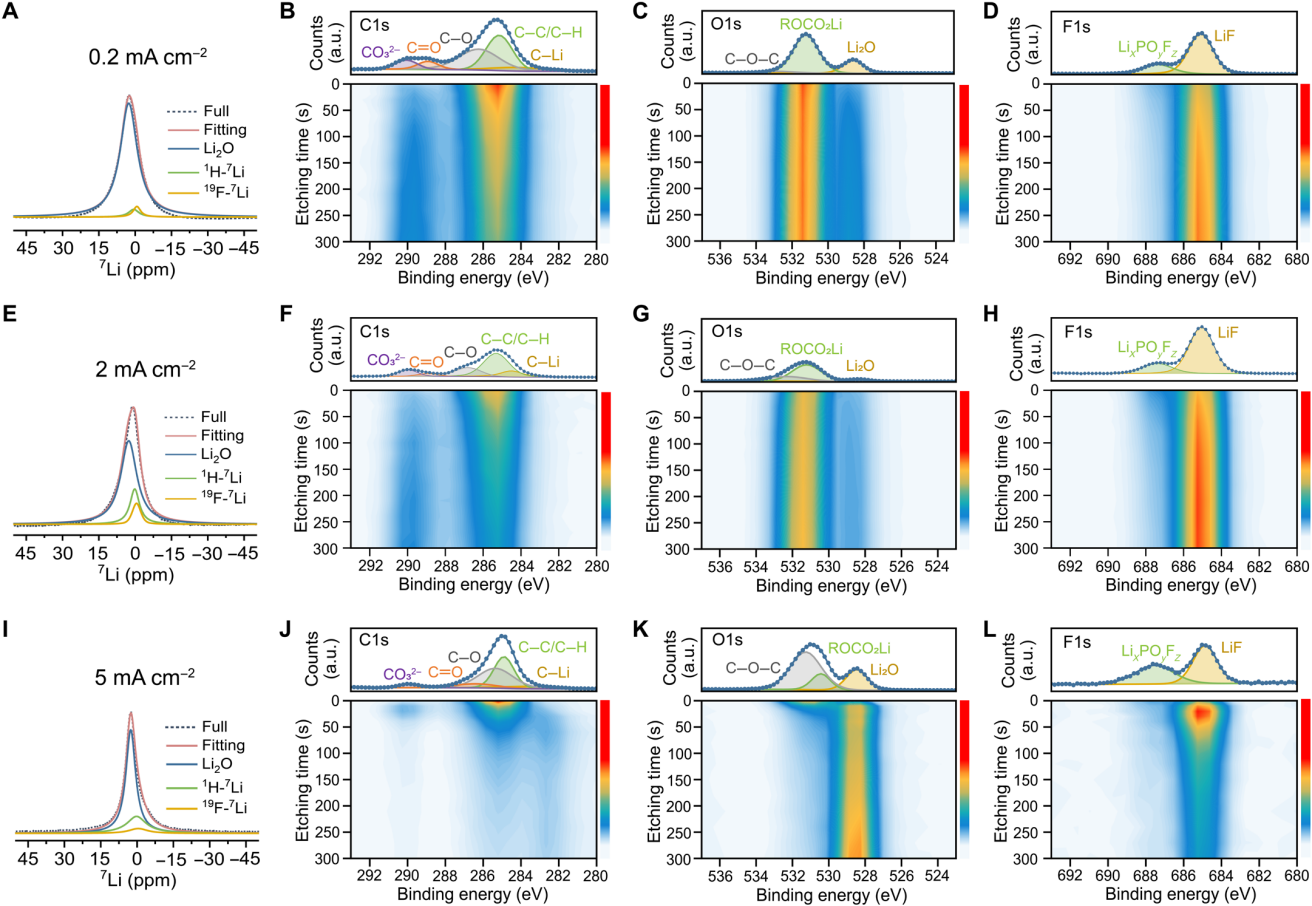
Determining the composition of the SEI on ED-Li initially plated at different current densities using ssNMR and XPS. SEI peak deconvolution for the plating current densities of (**A**) 0.2 mA cm^−2^, (**E**) 2 mA cm^−2^, and (**I**) 5 mA cm^−2^ by combining the fitting of the CP ssNMR spectra of ^1^H→^7^Li (Li near H), ^19^F→^7^Li (Li near F), and one-pulse measurement of bulk Li_2_O. Depth-profiled XPS measurements of C1s, O1s, and F1s for the ED-Li plated on Cu at (**B** to **D**) 0.2 mA cm^−2^, (**F** to **H**) 2 mA cm^−2^, and (**J** to **L**) 5 mA cm^−2^. Each plot comprises of two individual figures, i.e., the point before etching (up) and depth profiling spectrum (down). Color bar indicates the intensity from weak to strong from bottom to top.

To understand the improved Li kinetics, including the charge transfer and Li-ion diffusivity in the SEI, with a formation current density of 2 mA cm^−2^, the as-formed SEI composition and its distribution are investigated. Because of the strong dipolar interactions and the limited ^7^Li chemical shift range of the mainly diamagnetic SEI species, it is difficult to discern the various Li-containing components by a direct ^6,7^Li excitation ssNMR measurement (fig. S12 shows the SEI spectrum for ^6^Li ssNMR). To deconvolute the spectra, CP MAS ssNMR experiments were carried out for ^1^H→^7^Li and ^19^F→^7^Li to resolve the resonances, where Li is in close spatial proximity to ^1^H and ^19^F, respectively (see the pulse sequence in fig. S13). In these two cases, ^1^H and ^19^F are considered as the abundant nuclei, and during the CP MAS experiment, transfer of magnetization takes place to any ^7^Li environment in the vicinity (few atomic bond lengths) during a specific time duration (contact time). The ^7^Li near ^1^H is more likely to be assigned to the presence of an organic phase in the SEI, i.e., oligomers/polymers, (CH_2_OCO_2_Li)_2_ or ROCO_2_Li, while the ^7^Li close to ^19^F is assigned to LiF or possible residual Li salt (*22*). Nonetheless, there is still a substantial proportion of other species in the SEI, likely oxygen-containing Li compounds such as Li_2_O, LiOH, or Li_2_CO_3_ (fig. S14 for single-pulse measurements of Li_2_O, LiOH, and Li_2_CO_3_). Apparently, the offset of SEI chemical shift from 0 ppm is mainly caused by the existence of Li_2_O; therefore, bulk Li_2_O was additionally added to fit the SEI spectra. By resolving the chemical shift and the full width at half maximum of these three components and keeping these constant while fitting the SEI spectra (see separate spectra of ^1^H→^7^Li and ^19^F→^7^Li for different current densities in fig. S15), the basic components of the SEI generated at different C-rates on ED-Li can be resolved (Fig. 4, A and E, and fig. S16A). The SEI for ED-2 contains considerably more LiF and H-containing Li species among all samples. This is expected as LiF has been regarded to be effective in inhibiting lithium dendrites and the decomposition of the electrolyte, and the H-containing phase has higher affinity to the organic electrolyte solvent, thus facilitating the transport of Li-ions through the SEI (*22*, *49*). What is worth noting is that the LiF peak for the ED-5 sample is broader compared to that for the other samples, which could be attributed to a more amorphous LiF phase, generated by a high current density. In addition, ED-2 has the smallest amount of Li_2_O (or in this case, the Li_2_O with other residual species in the SEI), while ED-0.2, ED-0.5, and ED-5 contain much more Li_2_O. The insulating nature of Li_2_O present in larger amounts of the SEI can be partially responsible for suppressing the Li-ion conduction inside the SEI (*31*, *50*). We can additionally use this spectral deconvolution to deduce which species within the SEI exchange Li-ions effectively with Li-metal, which is shown in the F2 projections in Fig. 2 (D to F). The Li correlated to ^1^H and ^19^F is given in green and purple and Li_2_O (plus residual Li-containing components) in blue. Given that Li_2_O forms the largest constituent inside the SEI, the exchange is dominated by the Li-ions in the Li_2_O phase and Li-metal phase. It is worth noting that the ED-2 sample shows strong cross-peak intensity but with the presence of a smaller amount of Li_2_O, indicating that the Li exchange is facilitated by Li species in the vicinity of an H-rich environment and Li near F in the SEI as well.

In addition, depth-profiling XPS was performed on the initially plated ED-Li on Cu foil to further deconstruct the SEI. C1s (Fig. 4, B, F, and J, and fig. S16B), O1s (Fig. 4, C, G, and K, and fig. S16C), and F1s (Fig. 4, D, H, and L, and fig. S16D) spectra are presented for ED-0.2, ED-2, ED-5, and ED-0.5, respectively. Consistent with the CP MAS NMR results (including the ^1^H→^13^C CP MAS results in fig. S17), the XPS results show that for the sample plated at 2 mA cm^−2^, the SEI contains less insulating organics and Li_2_O species and larger fraction of LiF as compared to all the other samples. The peak in the F1s spectrum at ~685 eV, attributed to LiF (*51*–*53*), is much higher for the ED-2 sample, which also confirmed in the 1D single-pulse ^19^F spectra in fig. S18, while the peak at ~529 eV, assigned to Li_2_O (*54*, *55*), is much weaker at this formation current density. For the ED-5 sample, the organic phase and the LiF tend to be more distributed on the surface of the SEI, while the Li_2_O intensity is much stronger and slowly increases with the etching level. The origin could be that the high C-rate introduces dense Li nucleation sites and thereby induces highly packed Li-metal deposits, which block the entry of the electrolyte; thus, the decomposition reaction mainly occurs at the surface (*13*, *23*, *39*). The higher Li_2_O signal is probably ascribed to the large current density, which induces more intense decomposition reactions between the bulk Li and the electrolyte solvent, and introduces a thicker SEI film. Although Li_2_O has been reported in several studies for fostering the Li-ion exchange (*16*, *56*), our observations indicate that too much Li_2_O is detrimental and impedes Li-ion kinetics between SEI and Li-metal; therefore, an optimum amount of Li_2_O appears critical, especially near the Li-metal surface. In general, we observe that for the same plating areal capacity (1 mAh cm^−2^), the intensity of the Li-containing SEI species increases with the current density (fig. S19), which corroborates that the high current density leads to denser deposition morphology (*23*, *57*).

The CP MAS ssNMR and XPS results present here reveal how the SEI components and their distribution are affected by current densities. At a reasonably high plating current density (2 mA cm^−2^), an anion-rich, i.e., LiF-rich, SEI is formed. Several studies have reported the relation between applied current densities and the SEI species and structure formed. At high rates, thicker and mosaic SEI structures are more likely to be formed, which implies that current density plays a crucial in regulating the SEI properties, yet other parameters such as the electrolyte chemistry still need to be taken into account (*8*, *14*, *22*, *55*).

### Quantification of the dead Li

**Fig. 5.**
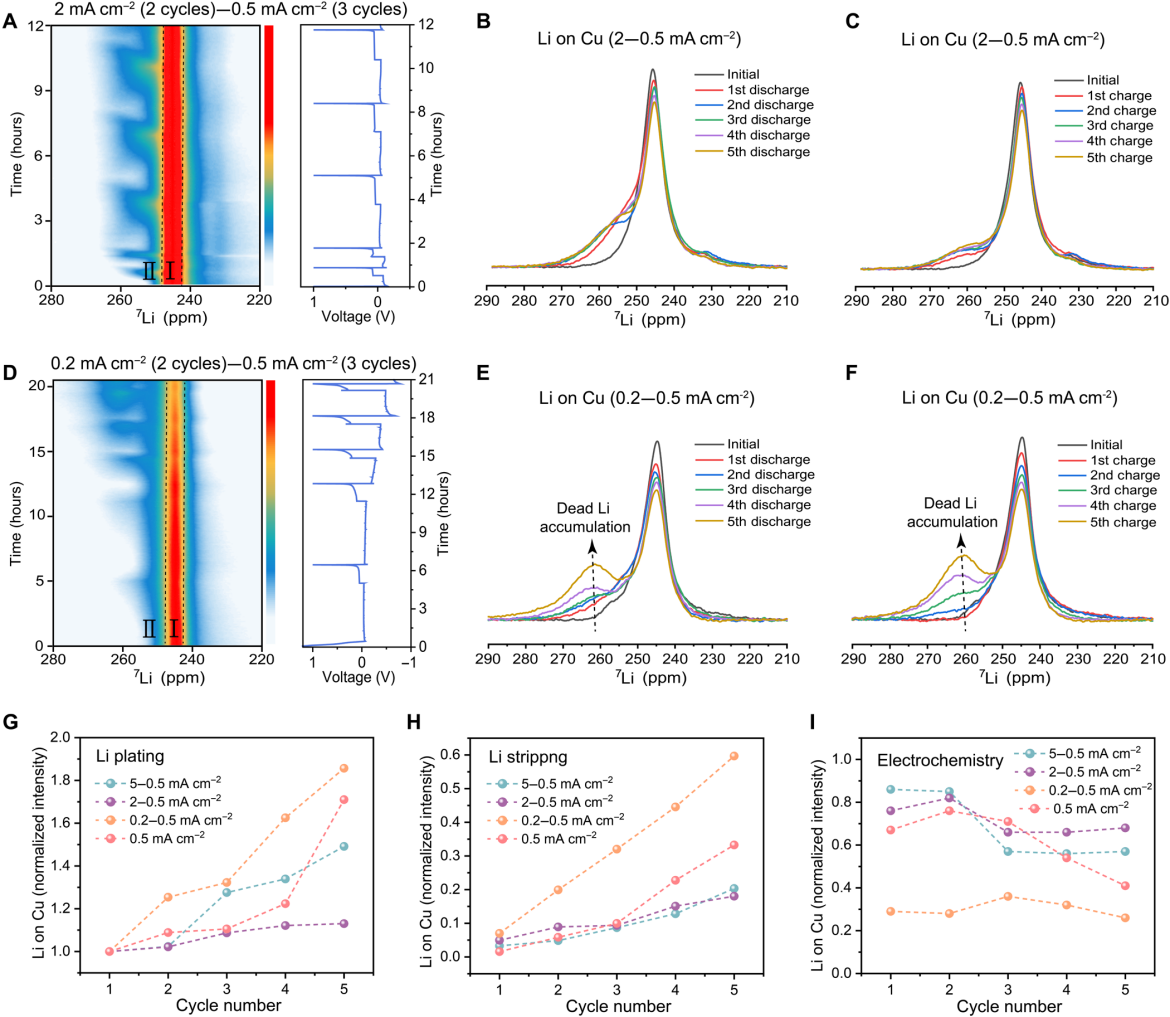
Operando NMR study of the evolution of dead Li for the Li||Cu cells cycled with different formation current densities. (**A** and **D**) contour plots of the ^7^Li NMR spectra acquired during the plating/stripping of Li-metal, along with the electrochemical voltage profile. Each cell was precycled at 2 or 0.2 mA cm^−2^, respectively, for two cycles and followed by three working cycles at 0.5 mA cm^−2^. The ^7^Li NMR signals at the end of discharge (**B** and **E**) and charge (**C** and **F**) for the five cycles, along with the initial Li peak are plotted separately. Normalized intensities of the Li signal on Cu at the end of (**G**) plating and (**H**) stripping (the intensities were normalized to the observed plated-Li signal at the end of the first discharge) and (**I**) CEs obtained from the electrochemistry for the five cycles of the cells cycled at 5 to 0.5 mA cm^−2^, 2 to 0.5 mA cm^−2^, 0.2 to 0.5 mA cm^−2^, and 0.5 mA cm^−2^. Color bar indicates the intensity from weak to strong from bottom to top.

With the aim of quantifying dead Li and SEI formation under the influence of varying formation current densities, operando ^7^Li NMR experiments are conducted and compared in four distinct electrochemical protocols, i.e., two formation cycles at 5, 2, 0.5, and 0.2 mA cm^−2^, respectively, followed by three working cycles at 0.5 mA cm^−2^, reaching a capacity of 1 mAh cm^−2^ for each charge step. Fig. 5 (A and D) and fig. S20 (A and D) show the contour plots of the operando ^7^Li NMR spectra together with the corresponding voltage profiles from the galvanostatic cycling. As displayed in these contour plots, all the cells form two co-existing Li-metal phases during the cycling. On the basis of the evolution of these phases, we can infer that phase I is from the Li-metal electrode (~245 ppm), and the emergence and diminishing of the Li signal of phase II directly correspond to the electrochemical plating and stripping of the Li-metal (247 to 270 ppm) (*58*–*61*). The largest Li chemical shift span of phase II is observed in the cell (0.2 to 0.5 mA cm^−2^), which arises from the inhomogeneous Li deposits in the formation cycles (also seen in the uneven Li plating from the SEM image in Fig. 1F). This subsequently leads to severe accumulation of dead Li-metal in the working cycles. The individual ^7^Li NMR spectra have been extracted at the end of each charge and discharge step, where the Li intensity for each plating (discharging) step is much more constant and reversible for the cell pretreated by 2 mA cm^−2^ than the 0.2 mA cm^−2^ one (Fig. 5, B and E), while as comparisons, the cell cycled at 5 and 0.5 mA cm^−2^ experiences an obvious build-up of Li-metal intensity over the cycles as well (fig. S20, B and E).

Observing that the resonance of the Li source overlaps to some extent with the signal of the plated Li on Cu, we decouple the Li on Cu resonance by fitting the individual components in each spectrum for the end of charge (stripping) shown in fig. S21. As expected, the cell precycled at 2 mA cm^−2^ shows the smallest increase in the Li-metal signal after stripping, indicating the smallest accumulation of dead Li (Fig. 5C and fig. S21B); however, the cell cycle with a small formation current density (0.2 mA cm^−2^) experiences pronounced dead Li accumulation, especially during the working cycles (Fig. 5F and fig. S21C), while the control group (cell activated and constantly cycled at 0.5 mA cm^−2^) shows fast growth of dead Li during cycling (figs. S20C and S21D). By contrast, the cell (5 to 0.5 mA cm^−2^) generates much more dead Li at the initial cycles already but with slower dead Li growth during following cycles (figs. S20F and S21A).

The normalized integrated area of the ^7^Li NMR peak of Li on Cu shows that the average plated Li signal detected by NMR (Li_NMR_) at the end of the fifth discharge (plating) has increased ~13% for the cycled sample of 2 to 0.5 mA cm^−2^ [(1.13 − 1) × 100% = 13%, normalized to the observed plated Li intensity at the end of the first discharge], where the accumulated dead Li accounts for roughly 18% of the initial charge Li signal at the end of the fifth charge (stripping) (Fig. 5, G and H, purple). At this point, the CE of 66 to 82% was realized over these five cycles (Fig. 5I, purple). In contrast, the cell using the cycling procedure of 0.2 to 0.5 mA cm^−2^ displays a rapid linear accumulation of dead Li with each cycle, reaching a dead Li ratio for almost 85% at the end of the fifth discharge, resulting in a low CE around 30% (Fig. 5, G to I, orange). Also, for the cell (5 to 0.5 mA cm^−2^), 49% dead Li was accumulated at the end of the fifth cycle, which corresponds to the low CE during the working cycles (Fig. 5, G to I, green). The stability of the cell cycled at continuous 0.5 mA cm^−2^ is somewhere in between (Fig. 5, G to I, red).

The efficiency of Li plating (lithium efficiency, LE_Li_) can be estimated from the inactive Li_NMR_ intensity, combined with the CE measured from electrochemistry (table S1), where the capacity loss caused by forming the SEI (SEI efficiency, CE_SEI_) can also be reckoned (see text S2) (*37*, *38*). The LE_Li_ for the cell cycled at 5 to 0.5 mA cm^−2^ protocol is relatively constant but showing a gradual downward trend in the working cycles, which is reflected in the decreasing CE and the accompanying growth in CE_SEI_ (fig. S22A). The LE_Li_ is constant and stabilizes at about 90% for the cell cycled at 2 to 0.5 mA cm^−2^, where in the later three working cycles, the CE is lower due to extra SEI formation (fig. S22B). Contrarily, most of the SEI was generated in the first two formation cycles for a formation current density of 0.2 mA cm^−2^, where the resulting Li aggregation is assumed to cause a sustained decline of LE_Li_ and consistently low CE over the cycles (fig. S22C). Also, the LE_Li_ of the cell cycled at 0.5 mA cm^−2^ are continuously declining with low CE_SEI_ (fig. S22D).

## DISCUSSION

The current density during the first formation cycles plays a critical role in controlling the properties of the initial SEI and Li deposition morphology, which strongly affects subsequent Li plating and stripping. To investigate this, ssNMR was performed to quantify the equilibrium Li-ion exchange between the electrochemically deposited Li-metal (ED-Li) and the as-formed SEI. This includes the charge transfer under equilibrium conditions, the exchange current density, and diffusivity within the SEI. The results demonstrate that the charge transfer kinetics of the initially formed SEI contribute substantially to the efficiency of the subsequent Li-metal plating and stripping cycles. From the range of formation current densities investigated (0.2, 0.5, 2, and 5 mA cm^−2^), the optimal cell performance was obtained when applying a relative high current density (2 mA cm^−2^), reflected by a stable CE of ~96% after 100 cycles in the standard 1 M LiPF_6_ in EC/DMC electrolyte (Fig. 1). Visualizing the Li-ion exchange between the ED-Li and the initial as-formed SEI phases with 2D-EXSY ssNMR experiments reveals an increase in the Li-ion exchange with increasing formation current densities (Fig. 2, C to F). The Li-ion exchange was quantified by 1D-EXSY ssNMR experiments, where the lowest *E*_a_ for exchange between SEI and Li-metal was found for 2 mA cm^−2^ (Fig. 3B), correlating to the highest CE. This is consistent with Xu *et al.* (*22*) who reported that Li||Cu cells have the lowest impedance at 2 mA cm^−2^ in the carbonate-based electrolyte system. This suggests that the exchange between Li-metal and SEI is a rate-limiting step during plating, dominating the formation of the Li-metal morphology. Moreover, the *E*_a_ for exchange reduces remarkably (from 44 up to 70%) upon subsequent cycling, indicating that the Li-ion exchange between Li-metal and SEI evolves, increasing upon subsequent cycling, thus improving ionic contact. This underlines that the SEI grown during the formation cycles has a lasting impact, also proposed by Lv *et al.* (*28*) and Oyakhire *et al.* (*14*), where the porous SEI established during formation acts as template for the Li-metal plating upon repeated cycling.

Comparing the ED-Li microstructures visualized from the SEM and TEM images (Fig. 1F and fig. S1), the more compact morphology, smaller particle size and thinner SEI for the sample directly plated at 2 mA cm^−2^ suggests homogenous Li-ion morphology. This is consistent with the results reported by Pei *et al.* (*23*) and Zhang *et al.* (*29*), where the authors concluded that more uniform and smaller Li nuclei generated at higher C-rate leads to a more homogeneous and planar Li deposition morphology. Our observation of better Li-ion exchange kinetics between the SEI and Li-metal, suggests better ionic contact at the optimal formation current density. CP MAS ssNMR and XPS (Fig. 4) indicate that the composition of the SEI is responsible for the different Li exchange kinetics, which is maximum for the lowest amount of Li_2_O and the largest amount LiF in the SEI (at 2 mA cm^−2^). When the current density reaches 5 mA cm^−2^, LiF is mainly distributed in the outer layer, which is consistent with the findings by Kanamura *et al.* (*62*). Although the roles of Li_2_O and LiF in the SEI are still debateable (*16*, *63*), the results presented here indicate that a relatively small amount of Li_2_O and larger amount of LiF in the SEI are beneficial for the Li-ion diffusion between the SEI and Li-metal, which is responsible for the improved reversibility of the cell. In addition, comparing the dead Li formation of the studied cycling protocols by the operando NMR experiments, the cell cycled at 2 to 0.5 mA cm^−2^ has the least dead Li build-up. The fast dead Li accumulation for the other cycling protocols could result from the impeded reversibility of Li deposition/stripping, caused by the high Li_2_O-containing poorly conductive SEI.

**Fig. 6.**
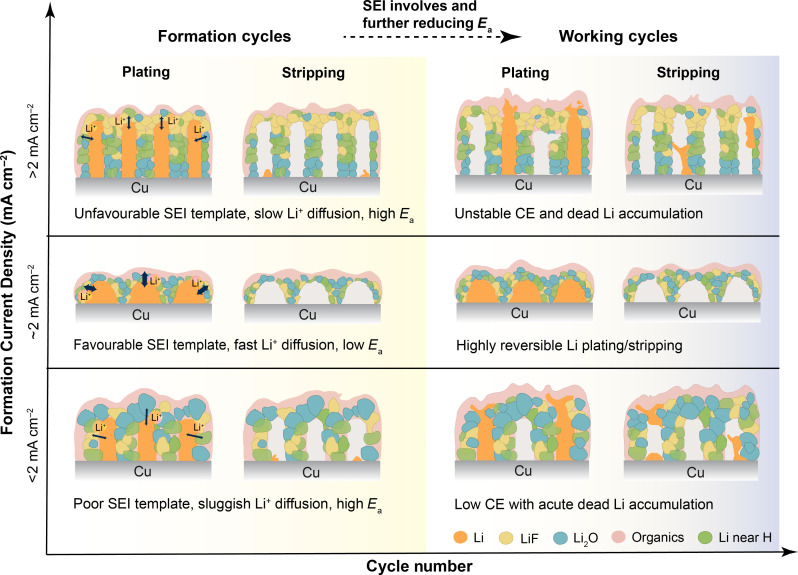
Formation current density-dependent SEI templates and their lasting impact on subsequent cycling.

As supported by the results above and schematically shown in Fig. 6, the initially formed SEI not only has a “template effect,” which can have a lasting impact on the subsequent cycles, but it also evolves during cycling, which is closely related to dead Li formation. By quantifying the fraction of dead Li-metal through operando NMR, we are able to correlate the SEI formation and dead Li formation with the different precycling protocols. Particularly, it certifies that the formation cycles contribute markedly to the overall cycling stability and the efficiency of Li-metal anodes. The irreversible capacity is mainly attributed to the as-formed SEI. This is consistent with the work reported by Gunnarsdóttir *et al.* (*37*). In all cases, there is SEI reformation and growth within each cycle; however, when first cycled with a higher current density (2 mA cm^−2^), the Li capacity loss toward the SEI formation is minimal, whereas it is nearly four times larger at lower formation current densities (0.2 mA cm^−2^; see fig. S22). For the subsequent working cycles, the CE is apparently higher and more stable. This further verifies that the formation cycles at different C-rates have a long lasting impact.

The different SEI formation rates arise from the distinct morphologies of the Li-metal deposits and the ionic pathways generated under different electrochemical conditions. Therefore, the Li-ion diffusion kinetics highly depend on the degree of passivation and surface area of the ED-Li and the as-formed SEI. During electrochemical preconditioning, the initial SEI forms a template that lasts (Fig. 6), in which Li nucleation can be assumed to take place on subsequent cycling, affecting the Li plating/stripping efficiency. Although increasing the current density is generally expected to favor a dendritic morphology, within the specific current range studied, an intermediate rate leads to denser Li nuclei distribution and smooth ED-Li surface because of the formation of a favorable SEI at this formation current density, which is beneficial for the long-term stability and reversibility of the LMBs (Fig. 6). This optimal C-rate can be expected to depend on the electrolyte system. For example, a smaller formation current density (0.5 mA cm^−2^) leads to the best cycling performance for the weakly solvating electrolyte, 1 M lithium bis(fluorosulfonyl)imide salt in fluorinated 1,4-dimethoxylbutane solvent (*14*). The Li-ion diffusion kinetics of the SEI and the lasting effect of formation cycles on the subsequent cycling have not been experimentally reported to the best of our knowledge. Combining the cell performance and the local Li-ion diffusion kinetics, the present results provide insight in how electrochemical preconditioning can affect the overall battery performance by influencing the chemical composition and distribution of the SEI.

In conclusion, a combination of various experimental techniques has been used to provide a dynamic-resolved and quantitative analysis of the lasting influence of the formation current density on Li-metal anodes. Combining ssNMR with SEM, TEM, and XPS, the chemical composition and microstructure of the as-formed SEI are revealed. The spontaneous diffusion of Li-ions between electrochemically deposited Li-metal and the as-formed SEI is disclosed, reflecting a large dependence on the formation current density. Applying a intermediate high formation current density (2 mA cm^−2^) leads to higher Li-ion mobility between SEI and Li-metal. The denser Li microstructure and favorable chemical SEI composition enable more homogeneous Li deposition and less inactive/dead Li formation. The lasting impact of this initial SEI facilitates Li-ion diffusion between the separate solid phases effectively, which creates ideal conditions for subsequent cycling and gives rise to great improvement of the stability and efficiency of the Li-metal anode. The present work provides in-depth understanding of the formation cycles in LMBs, where the spontaneous Li-ion diffusion dynamics between Li-metal and the SEI can be a potential guide in conjunction with other battery optimization strategies, such as electrolyte design, current collector engineering, and new electrode materials development. Beyond that, the present work could be further extended to solid-state electrolyte systems and other redox chemistries.

## MATERIALS AND METHODS

### Batteries assembly and electrochemical testing

The Li||Cu cells were assembled with lithium disk (~500 μm thick; Sigma-Aldrich) and Cu foil (~11 μm thick; >99.99%; MTI Corporation) as electrodes and a polyethylene (PE) (Celgard 2300) separator and a piece of glass fiber (Whatman GF/D) as separators (after being dried in vacuum at 80°C for more than 24 hours). The electrolytes used were (i) conventional carbonate-based 1 M LiPF_6_ in EC/DMC [1:1 (w/w). Sigma-Aldrich], (ii) 1 M LiPF_6_ in EC/DMC [1:1 (w/w)] with 10 wt % FEC and 1 wt % VC (Dodochem Co., Ltd.), (iii) 1 M lithium LiTFSI in 1,2-DME and DOL [1 M LiTFSI in DOL/DME; 1:1 (w/w); Dodochem Co. Ltd.], and (iv) 1 M LiTFSI in DOL/DME [1:1 (w/w)] with 5 wt % LiNO_3_ (Dodochem Co. Ltd.). Cell assembly, disassembly, and handling of air sensitive materials were done in an argon-filled glovebox (O_2_ < 1 ppm, H_2_O < 0.1 ppm).

Galvanostatic cycling was performed on a Maccor 4000 battery cycler or a Land CT2001A at room temperature by deposition of Li onto the Cu working electrode with different current densities up to a total capacity of 1 mAh cm^−2^, followed by Li stripping at different current densities up to 1 V. The EIS measurements were obtained using an Autolab PGSTAT302N in the frequency range of 1 MHz to 0.1 Hz with a sinusoidal signal with Vrms = 10 mV. The EIS spectra were fitted with an equivalent circuit (EC) model by RelaxIS 3, where *R*_SEI_ is the SEI resistance, *R*_ct_ is the charge-transfer resistance, CPE is constant phase element, the respective CPE describes the capacitance of the corresponding process, and *W* is Warburg diffusion term.

### Solid-state NMR measurements

Individual samples were prepared by plating Li on Cu foil with different current densities (0.2, 0.5, 1, 2, and 5 mA cm^−2^) to a total amount of Li to 10 mAh cm^−2^ (electrodeposited samples) or plated to 10 mAh cm^−2^ at 0.5 mA cm^−2^ after a specific cycling procedure (cycled samples). After plating, the cells were disassembled in the glovebox and the plated electrodes were washed by dipping into DMC solvent for three to five times to remove residual electrolyte, and then the electrodes were dried in a vacuum chamber to evaporate the solvent. The ED-Li and SEI were removed from the Cu foil using a razor blade and mixed with KBr (dried in vacuum for 2 weeks at 80°C before bringing into glovebox) using a mortar and pestle in the glovebox to limit peak broadening caused by the paramagnetic nature of lithium and reduce eddy currents (*64*). The mixture was transferred into the 3.2-mm rotor and sealed with a Vespel cap.

All solid-state NMR measurements were performed on a Bruker Ascend 500-MHz magnet (B0 = 11.7 T) with an NEO console operating at frequencies of 194.37 MHz for ^7^Li, 73.6 MHz for ^6^Li, 500.130 MHz for ^1^H, 470.385 MHz for ^19^F, and 125.758 MHz for ^13^C. ^6,7^Li chemical shifts were referenced with respect to a 0.1 M LiCl solution (0 ppm), ^1^H and ^13^C chemical shifts were referenced in regard to solid adamantane (^1^H at 1.81 ppm and ^13^C at 38.48 ppm), and ^19^F was referenced to LiF at 204 ppm. A Bruker three-channel MAS 3.2-mm direct VT (DVT) probe was used for all the measurements. The samples were filled into 3.2-mm zirconia rotors, and a MAS frequency of 15 kHz was applied.

One-pulse ^7^Li, ^6^Li, and ^19^F experiments were performed with π/2 pulse lengths of 4.76, 6, and 3.05 μs, respectively. A recycle delay of about four times *T*_1_ was used for each nuclei, where the *T*_1_ was determined using saturation recovery experiments. ^7^Li-^7^Li 2D-EXSY measurements were performed for these samples at a mixing time of 150 ms. All 2D spectra consisted of eight scans for each of the 1950 transients, and each transient was incremented by 6.67 μs.

VT ^7^Li 1D-EXSY measurements were performed with a recycle delay of 8 s per mixing time (*T*_mix_), and *T*_mix_ was varied between 0.01 ms and 0.1 s with totally 16 time intervals (since the exchange reaches the maximum before *T*_mix_ = 0.06 s, the normalized intensities of *T*_mix_ > 0.06 s are reduced in order to obtain better fitting). The pulse sequence with the appropriate phase cycle for cancelation of direct magnetization that may occur after *T*_1_ relaxation has been described in detail elsewhere (*44*, *47*), which consists of π/2, τ, π, τ, −π/2, *T*_mix_, +π/2, and acquisition, with π/2 pulse length = 5.4 μs. An echo time τ ranging from 200 to 800 μs was used to filter out the SEI resonance with a short *T*_2_, effectively functioning as a *T*_2_ filter. For each sample, these ^7^Li 1D-EXSY experiments were performed at five different temperatures (283, 298, 313, 328, and 343 K).

^19^F→^7^Li CP MAS experiments were performed with a radio frequency (rf) field strengths of 82 kHz and a ramped (90 to 100%) amplitude of ^19^F during CP. A contact time of 200 μs was used, with a recycle delay of 2 s and between 1024 and 2048 scans. For ^1^H→^7^Li CP MAS experiments, an rf field strengths of 81 kHz and contact times of 3 ms were used, the rf field amplitude of ^1^H during CP experiments was ramped from 70 to 100%, and 10,240 scans were acquired for each sample with a recycle delay of 1 s. The ^1^H→^13^C CPMAS experiments were measured with an initial ^1^H π/2 pulse of 3.86 μs. During the CP measurements for ^13^C, an rf field strength of 64 kHz was used and 10,240 scans were acquired for each sample with a recycle delay of 10 s.

### Operando NMR measurements

Li-metal disk of 0.4 × 0.7 (cm^2^) and Cu foil of 0.5 × 1 (cm^2^) were used to assemble the operando Li||Cu half-cells. One Celgard 2300 separator and one piece of glass fiber (Whatman GF/D) were used as separators. A 1 M LiPF_6_ in EC/DMC [1:1 (w/w)] electrolyte (25 μL) was added in the cells. Operando NMR experiments were conducted at ambient temperature on a Bruker Avance 500 MHz using a solenoidal Ag-coated Cu coil, which was synchronized with the external electrochemical cycler. The spectra were recorded using an in situ automatic tuning and matching probe (ATM VT X in situ WB NMR probe, NMR Service) that allows for an automatic recalibration of the NMR rf circuit during an operando electrochemical measurement. To quantify the operando NMR signal, the automatic retuning of the rf circuit is essential because the sample conditions keep changing during the electrochemical process. The probe is equipped with highly shielded wire connections to the electrochemistry with low-pass filters (5 MHz) attached to the probe, minimizing the interferences between the NMR and the electrochemical test circuit, details are described elsewhere (*65*). Single-pulse experiments were used to collect the operando NMR spectra, with a recycle delay of 2 s and 256 transients recorded. This leads to an experimental time of each spectrum for about 4.5 min. The shift of ^7^Li was internally referenced to Li-metal at 245 ppm. The acquired series of spectra were processed in the Bruker TopSpin software using the phase and baseline correction. Further data processing was done in MestReNova 11.0.

### SEM, XPS, and TEM characterizations

For SEM imaging, the samples were transferred into an SEM (JEOL JSM-6010LA) machine under dry Argon conditions by an air-tight Ar-filled sample holder. The images were taken using an accelerating voltage of 10 kV (secondary electron). XPS measurements were performed with a Thermo Fisher K-Alpha spectrometer to investigate the chemical compositions of the plating electrodes. The samples were transferred into the XPS machine under vacuum using an air-tight sample holder. The spectrometer is equipped with a focused monochromatic Al kα source (1486.6 eV) anode operating at 36 W (12 kV, 3 mA) and a flood gun operating at 1 V (100 μA). The base pressure of the analysis chamber was approximately 2 × 10^−9^ mbar, and the spot size was approximately 800 μm by 400 μm. Depth profiling was performed with an Ar^+^ ion gun operated at 3 kV, corresponding to 0.5 nm s^−1^ as calibrated on Ta_2_O_5_, and a pass energy of 200 eV was used. In the analysis, the binding energy was corrected for the charge shift relative to the primary C1s hydrocarbon peak at BE = 284.8 eV. For each sample, at least three points were measured, which showed similar results. The data were fitted using 70% Gaussian and 30% Lorentzian line shapes (weighted least squares fitting method) and nonlinear Shirley-type background using the Thermo Fisher Avantage software. TEM experiments were performed on TEM (Tecnai G2, FEI, USA) operated at 60 kV.

For both the SEM and XPS characterizations, the Li-metal–plated electrodes were prepared by charging the Li||Cu half cells to a capacity of 1 mAh cm^−2^ at variable current densities. The Cu foils with plated Li were rinsed with DMC for three to five times to remove the residual electrolyte in the glove box under a dry argon atmosphere and dried in a vacuum chamber. For the TEM experiments, a lacey carbon TEM grid was put on a Cu foil working electrode and assembled into Li||Cu cells in an argon-filled glovebox. The cells were charged to 1 mAh cm^−2^ capacity at variable current densities, and then the TEM grid was taken out by disassembling the cells for measurement. The TEM grid was carefully transferred into the TEM holder in the glovebox with a specialized shutter to prevent air exposure.

## Supplementary Materials

### This PDF file includes:

Texts S1 and S2 Figs. S1 to S22 Table S1
