# Supplementary material for: The lasting impact of formation cycling on the Li-ion kinetics between SEI and the Li-metal anode and its correlation with efficiency
Source: Sci Adv. 2024 Jan 17;10(3):eadj8889. doi: 10.1126/sciadv.adj8889 (PMC10793961; doi:10.1126/sciadv.adj8889)
Supplement: Supplementary file 1 — Texts S1 and S2 Figs. S1 to S22 Table S1 [file sciadv.adj8889_sm.pdf]

Science Advances
AAAS

# Supplementary Materials for

## The lasting impact of formation cycling on the Li-ion kinetics between SEI and the Li-metal anode and its correlation with efficiency

Shengnan Zhang *et al.*

Corresponding author: Ming Liu, liuming@sz.tsinghua.edu.cn; Swapna Ganapathy, s.ganapathy@tudelft.nl; Marnix Wagemaker, m.wagemaker@tudelft.nl

*Sci. Adv.* **10**, eadj8889 (2024)
DOI: 10.1126/sciadv.adj8889

**This PDF file includes:**

Texts S1 and S2
Figs. S1 to S22
Table S1

# Supplementary Texts

## Text 1: Quantification of interphases exchange

Quantification of exchange between the electrodeposited Li metal and the as-formed SEI was performed by fitting the growing SEI signal to a diffusion model where in a solution to Fick's law for diffusion is determined $\frac{\partial m(\vec{r},\vec{t})}{\partial t} = \vec{\nabla} \cdot \{D(\vec{r})m(\vec{r},\vec{t})\}$ , where $m(\vec{r},\vec{t})$ is the magnetization of Li at position $\vec{r}$ and t, and $D$ is the Li-ion self-diffusion coefficient. By using the mathematical models of Schmidt-Rohr and co-workers for spin diffusion, and by assuming the overall diffusivity to be equal to the effective diffusion coefficient, the rate of demagnetization of Li-ions in the Li metal can be set equal to the initial magnetization minus the rate of magnetization in the SEI. Assuming a SEI phase embedded in an infinite Li metal phase, this leads to the following analytical expression for the rate of demagnetization from the Li metal into SEI as

$$\mathrm{m}(t_{mix}) = 1 - \left\{\frac{m_0}{2}\sqrt{4Dt_{mix}}\left[ierfc\left(\frac{d}{\sqrt{4Dt_{mix}}}\right) + ierfc\left(\frac{-d}{\sqrt{4Dt_{mix}}}\right) - \frac{2}{\sqrt{\pi}}\right]\right\}^3$$

where $\mathrm{ierfc(x)} = 1/\sqrt{\pi}\,\exp(-x^2) - x[l - \mathrm{erf}\,(x)]$ and $d$ is the Li diffusion distance from the SEI phase to the Li metal.

## Text 2: Calculation of SEI efficiency and Li efficiency

The capacity loss of the electrochemistry originating from the SEI formation and inactive Li accumulation, therefore, the capacity loss caused by forming SEI can be estimated from the NMR detected inactive (dead) Li.

Coulombic efficiency ($CE$) from the electrochemical process is given by:

$$CE = \frac{C_{stripping}}{C_{plating}}$$

Where $C_{plating}$ is the capacity obtained by discharging the Li||Cu cell under specific current densities till a total capacity of 1 mAh cm$^{-2}$. $C_{stripping}$ is the capacity obtained by charging the cell to a cut-off voltage of 1 V.

Therefore the capacity loss $CE_{loss}$ from the electrochemistry is:

$$CE_{loss} = 1 - CE = CE_{dead\ Li} + CE_{SEI}$$

Where $CE_{dead\ Li}$ is the capacity loss resulted from the generation of irreversible Li, $CE_{SEI}$ is the capacity loss caused by SEI formation.

Therefore, $CE_{dead\ Li}$ and $CE_{SEI}$ for cycle n (n = 1, 2, 3, 4, 5, DC = discharge (plating), C = charge (stripping)) are:

$$CE_{dead\ Li,\ n} = \frac{Li_{end\ of\ C,\ n} - Li_{end\ of\ C,n-1}}{Li_{end\ of\ DC,n} - Li_{end\ of\ C,n-1}}$$

$$CE_{SEI,\ n} = CE_{loss,\ n} - CE_{dead\ Li,\ n}$$

Where $Li_{end\ of\ C,\ n}$ is the NMR detected Li signal at the end of charge (stripping) of the cycle n, $Li_{end\ of\ DC,\ n}$ is the NMR detected Li signal at the end of discharge (plating) of the cycle n.

The Li efficiency ($LE_{Li}$) during cycle n is:

$$LE_{Li,\ n} = 1 - CE_{dead\ Li,\ n}$$

This Li efficiency is obtained based on the dead Li signal detected by NMR. The difference between the $LE_{Li}$ and the $CE$ is the capacity loss caused by SEI formation,[1,2] then it leads to:

$$LE_{Li,\ n} = CE_n + CE_{SEI,\ n}$$

Take the first cycle of the 2-0.5 mA $cm^{-2}$ cell as an example. Based on the electrochemical test (**Fig. 5A**), the $CE$ for the first cycle is:

$$\mathrm{CE} = 76\%$$

$$CE_{loss} = 1 - CE = 24\%$$

According to the operando $^{7}$Li NMR measurement, :

$$CE_{dead\ Li} = \frac{Li_{end\ of\ C}}{Li_{end\ of\ DC}} = \frac{0.049}{0.416} \times 100\% = 11.8\%$$

(0.049 and 0.416 are the normalized intensities of the Li signal on Cu at the end of charge and discharge of the first cycle.)

$$CE_{SEI} = CE_{loss} - CE_{dead\ Li} = 24\% - 11.8\% = 12.2\%$$

The $LE_{Li}$ of the first cycle is:

$$LE_{Li} = 1 - CE_{dead\ Li} = 100\% - 11.8\% = 88.2\%$$

## Supplementary Figures

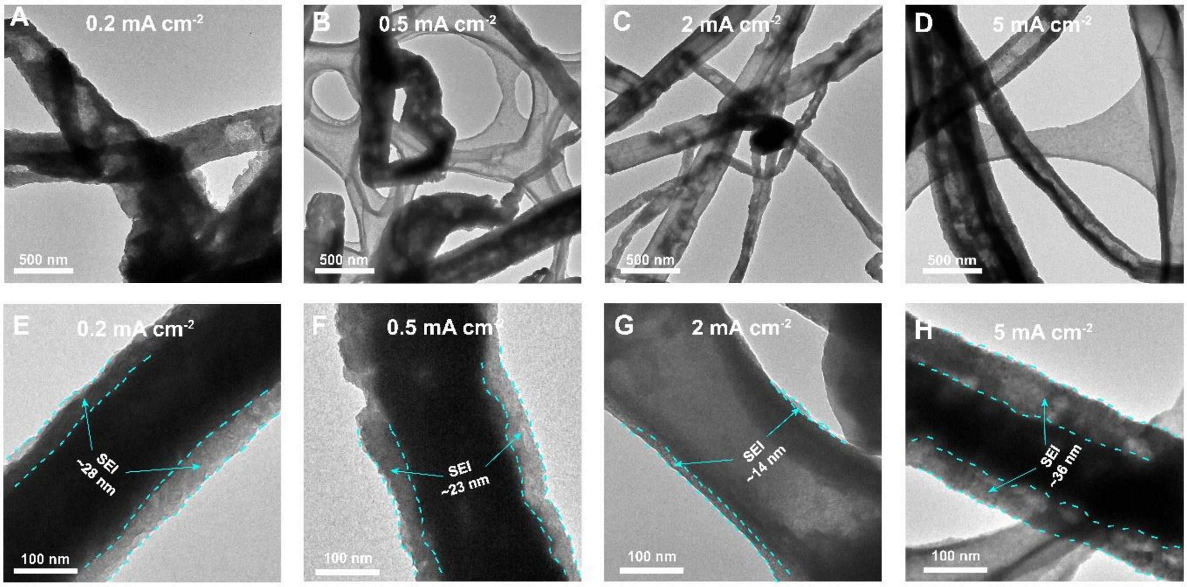


**Fig. S1**. **Bright field TEM images of the ED-Li and the SEI plated at different current densities.** (**A** and **E**) 0.2 mA $cm^{-2}$, (**B** and **F**) 0.5 mA $cm^{-2}$, (**C** and **G**) 2 mA $cm^{-2}$ and (**D** and **H**) 5 mA $cm^{-2}$. With dotted lines marking the SEI in (**E)** to (**H)**.

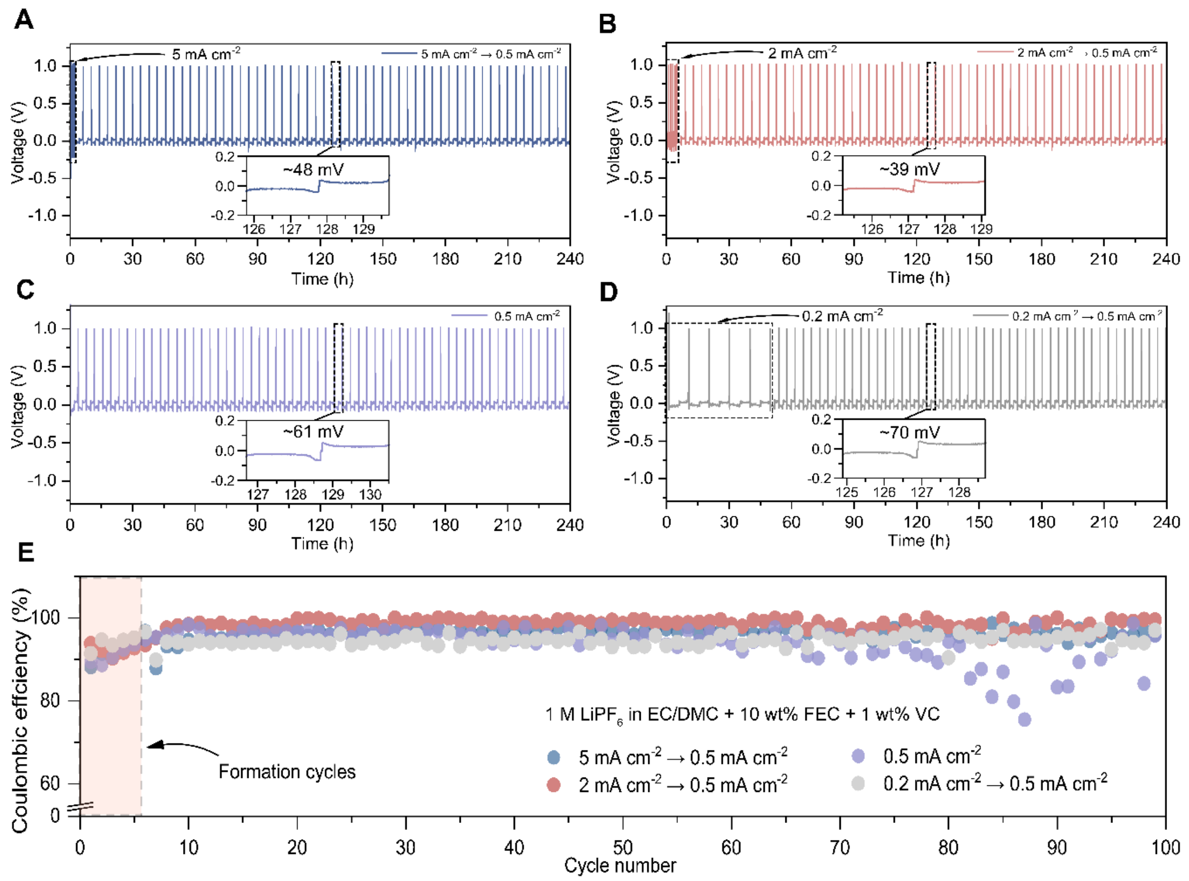


**Fig. S2. Electrochemical characterization of the LMBs cycled with different formation current densities using 1 M $LiPF_6$ in EC/DMC + 10 wt% FEC + 1 wt% VC electrolyte.** Galvanostatic cycling performance and polarization profiles of the Li||Cu cells cycled at: (**A**) 5 mA $cm^{-2}$ - 0.5 mA $cm^{-2}$, (**B**) 2 mA $cm^{-2}$ - 0.5 mA $cm^{-2}$, (**C**) 0.5 mA $cm^{-2}$ and (**D**) 0.2 mA $cm^{-2}$ - 0.5 mA $cm^{-2}$. Five formation cycles were applied in advance and Li was electrodeposited to a capacity of 1 mAh $cm^{-2}$ for each plating step, inserts are the enlarged view of the voltage profiles at 125~131 h indicating the overpotential values. (**E**) Comparison of the corresponding Coulombic efficiency for the Li||Cu cells cycled at the current densities given in (**A**) to (**D**).

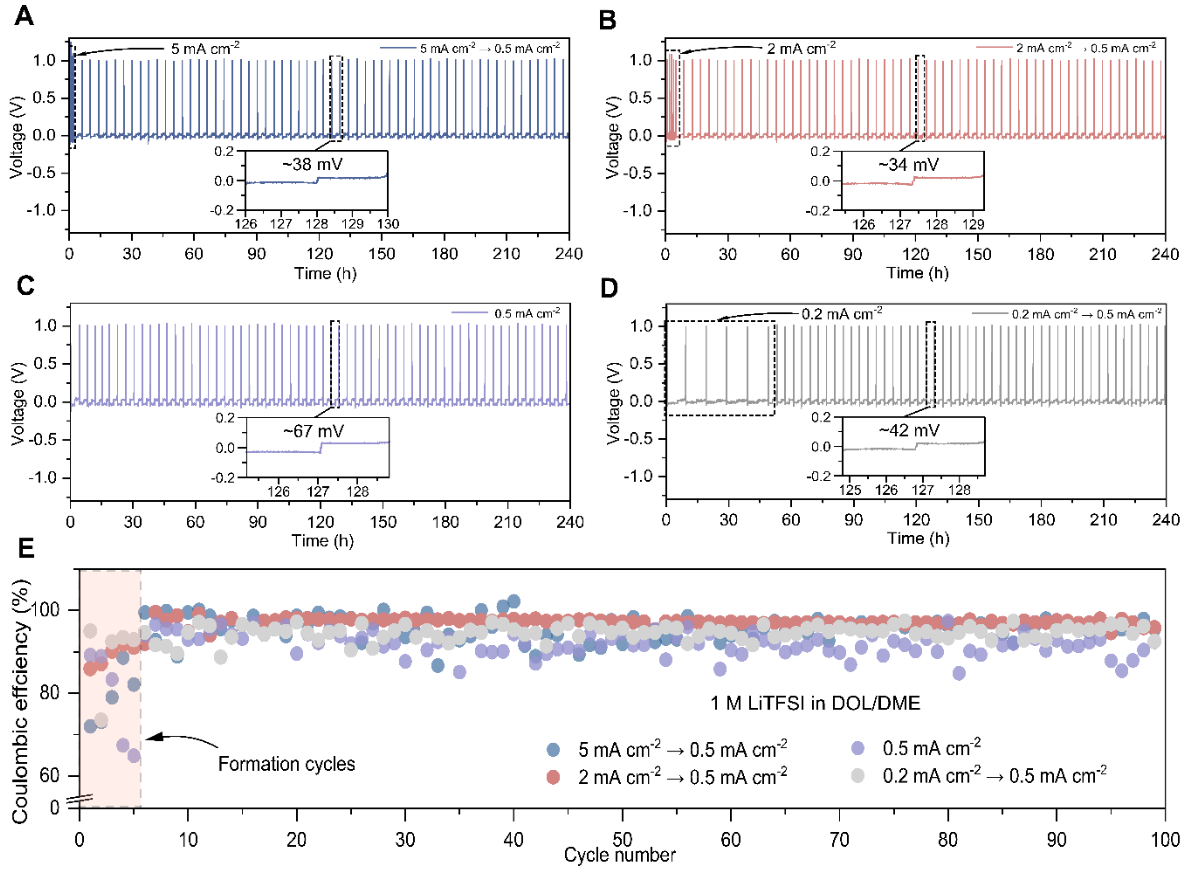


**Fig. S3**. **Electrochemical characterization of the LMBs cycled with different formation current densities using 1 M LiTFSI in DOL/DME electrolyte.** Galvanostatic cycling performance and polarization profiles of the Li||Cu cells cycled at: (**A**) 5 mA $cm^{-2}$ - 0.5 mA $cm^{-2}$, (**B**) 2 mA $cm^{-2}$ - 0.5 mA $cm^{-2}$, (**C**) 0.5 mA $cm^{-2}$ and (**D**) 0.2 mA $cm^{-2}$ - 0.5 mA $cm^{-2}$. Five formation cycles were applied in advance and Li was electrodeposited to a capacity of 1 mAh $cm^{-2}$ for each plating step, inserts are the enlarged view of the voltage profiles at 125~130 h indicating the overpotential values. (**E**) Comparison of the corresponding Coulombic efficiency for the Li||Cu cells cycled at the current densities given in (**A**) to (**D**).

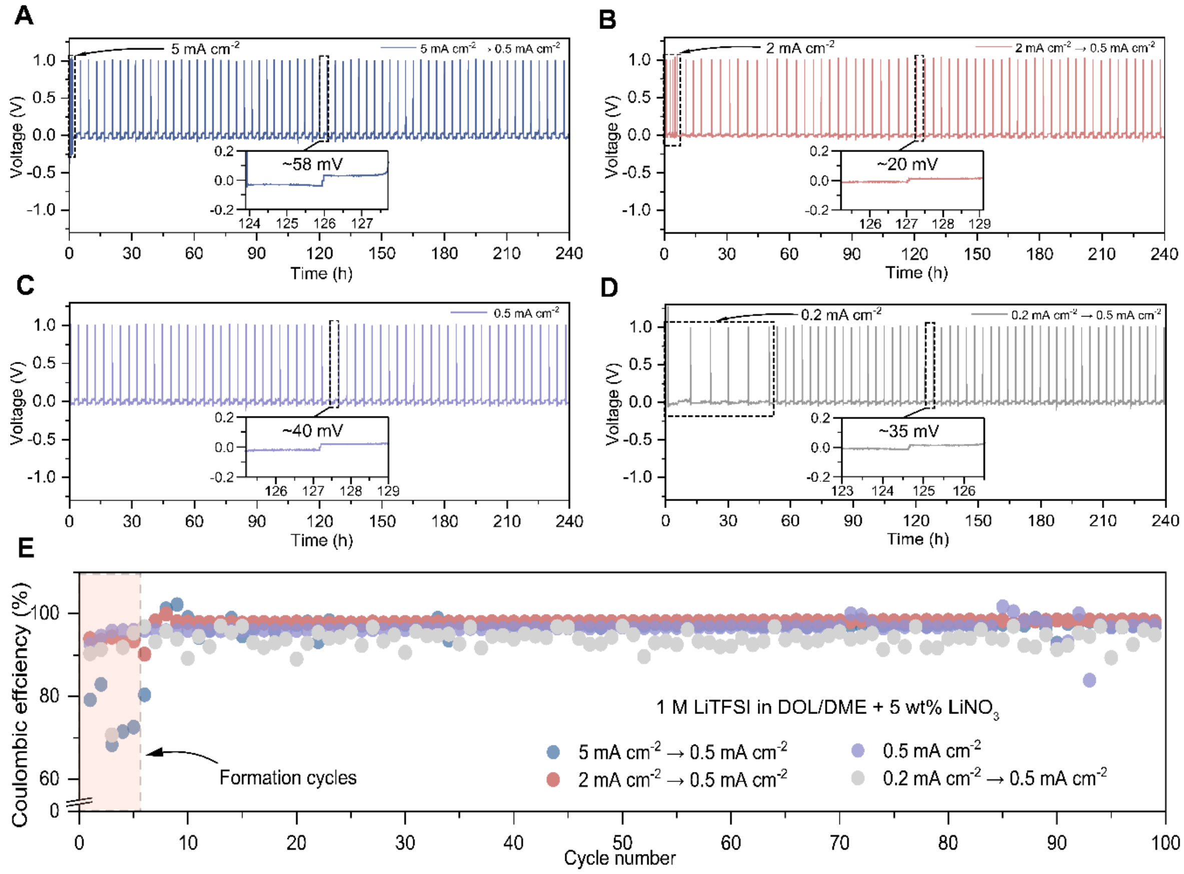


**Fig. S4. Electrochemical characterization of the LMBs cycled with different formation current densities using 1 M LiTFSI in DOL/DME + 5 wt% $LiNO_3$ electrolyte.** Galvanostatic cycling performance and polarization profiles of the Li||Cu cells cycled at: (**A**) 5 mA $cm^{-2}$ - 0.5 mA $cm^{-2}$, (**B**) 2 mA $cm^{-2}$ - 0.5 mA $cm^{-2}$, (**C**) 0.5 mA $cm^{-2}$ and (**D**) 0.2 mA $cm^{-2}$ - 0.5 mA $cm^{-2}$. Five formation cycles were applied in advance and Li was electrodeposited to a capacity of 1 mAh $cm^{-2}$ for each plating step, inserts are the enlarged view of the voltage profiles at 125~130 h indicating the overpotential values. (**E**) Comparison of the corresponding Coulombic efficiency for the Li||Cu cells cycled at the current densities given in (**A**) to (**D**).

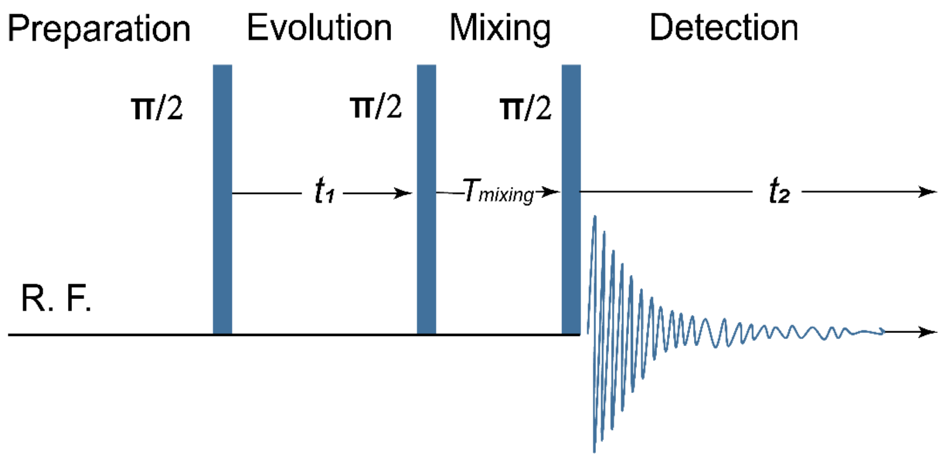


**Fig. S5**. **Schematic showing the pulse sequence employed for the 2D-EXSY ssNMR experiments.**

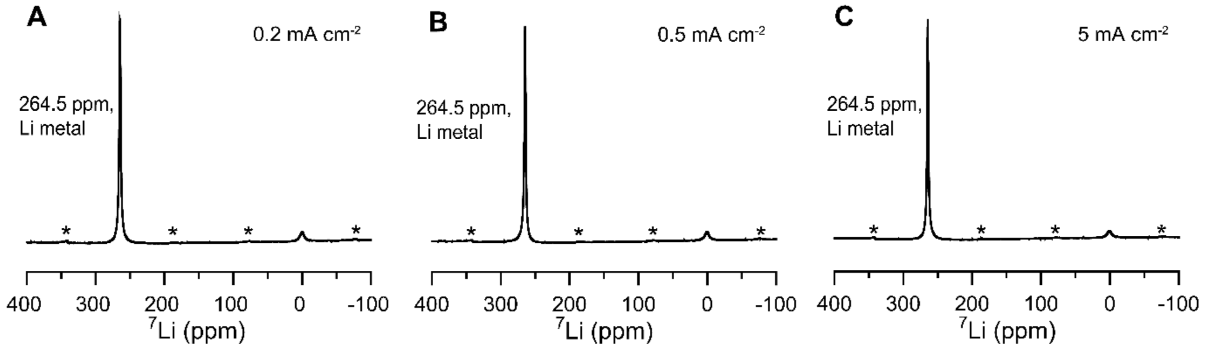


**Fig. S6**. **One-dimensional (1D) $^{7}$Li magic angle spinning (MAS) spectra of the ED-Li and the SEI.** (**A**) 0.2 mA cm$^{-2}$, (**B**) 0.5 mA cm$^{-2}$ and (**C**) 5 mA cm$^{-2}$. Asterisks denote spinning sidebands.

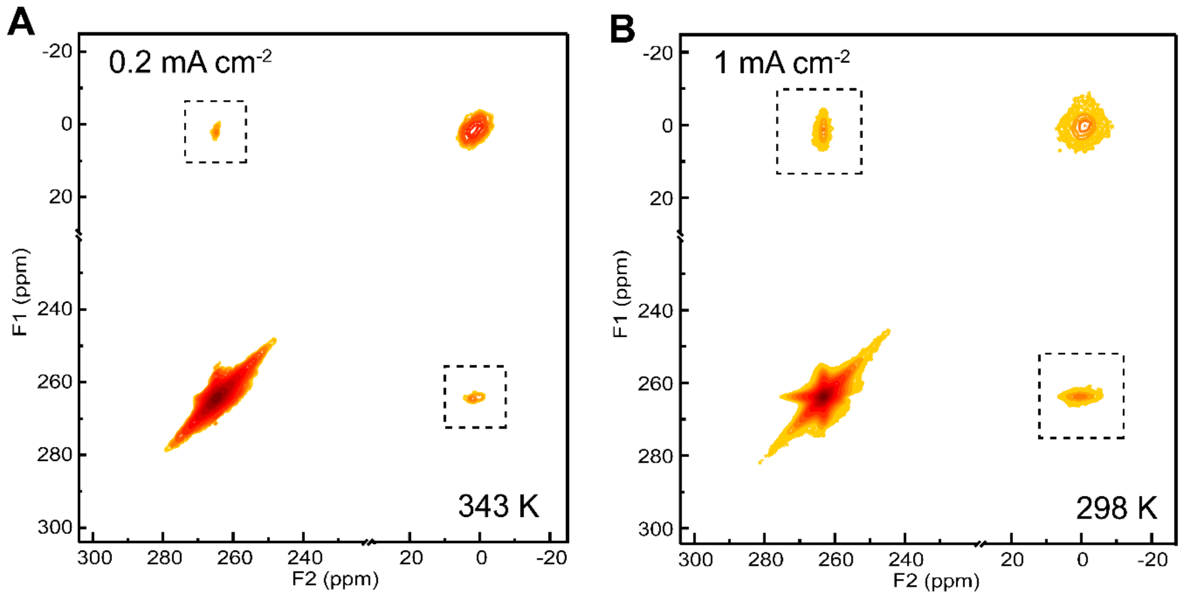


**Fig. S7**. **$^7$Li-$^7$Li 2D ssNMR exchange experiments measuring Li-ion transport between the electrodeposited Li (ED-Li) and the as-formed SEI.** Two-dimensional $^7$Li-$^7$Li exchange spectroscopy (2D-EXSY) of the ED-Li and the SEI formed at: (**A**) 0.2 mA cm$^{-2}$ (ED-0.2) and (**B**) 1 mA cm$^{-2}$ (ED-1) at $T_{mix}$ = 150 ms, among them the ED-0.2 was measured at 343 K, the ED-1 was measured at 298 K.

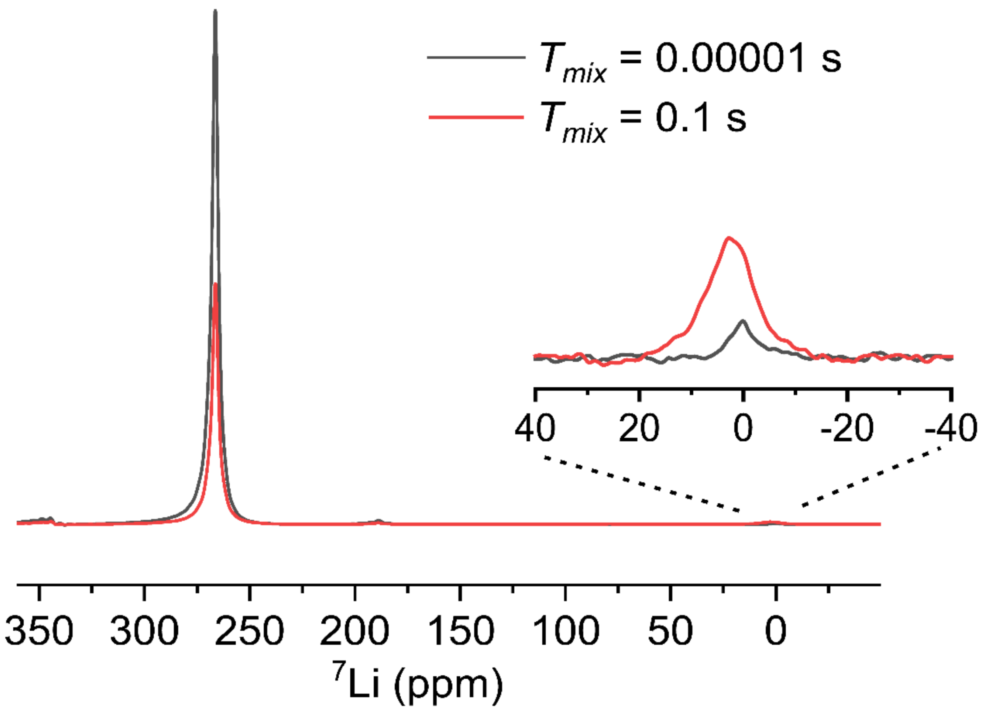


**Fig. S8**. **1D spectra of the first ($T_{mix}$ = 0.00001s) and last ($T_{mix}$ = 0.1s) slices of the one-dimensional $^{7}$Li exchange spectroscopy (1D-EXSY) experiment.** Sample shown here is ED-2 at 298 K.

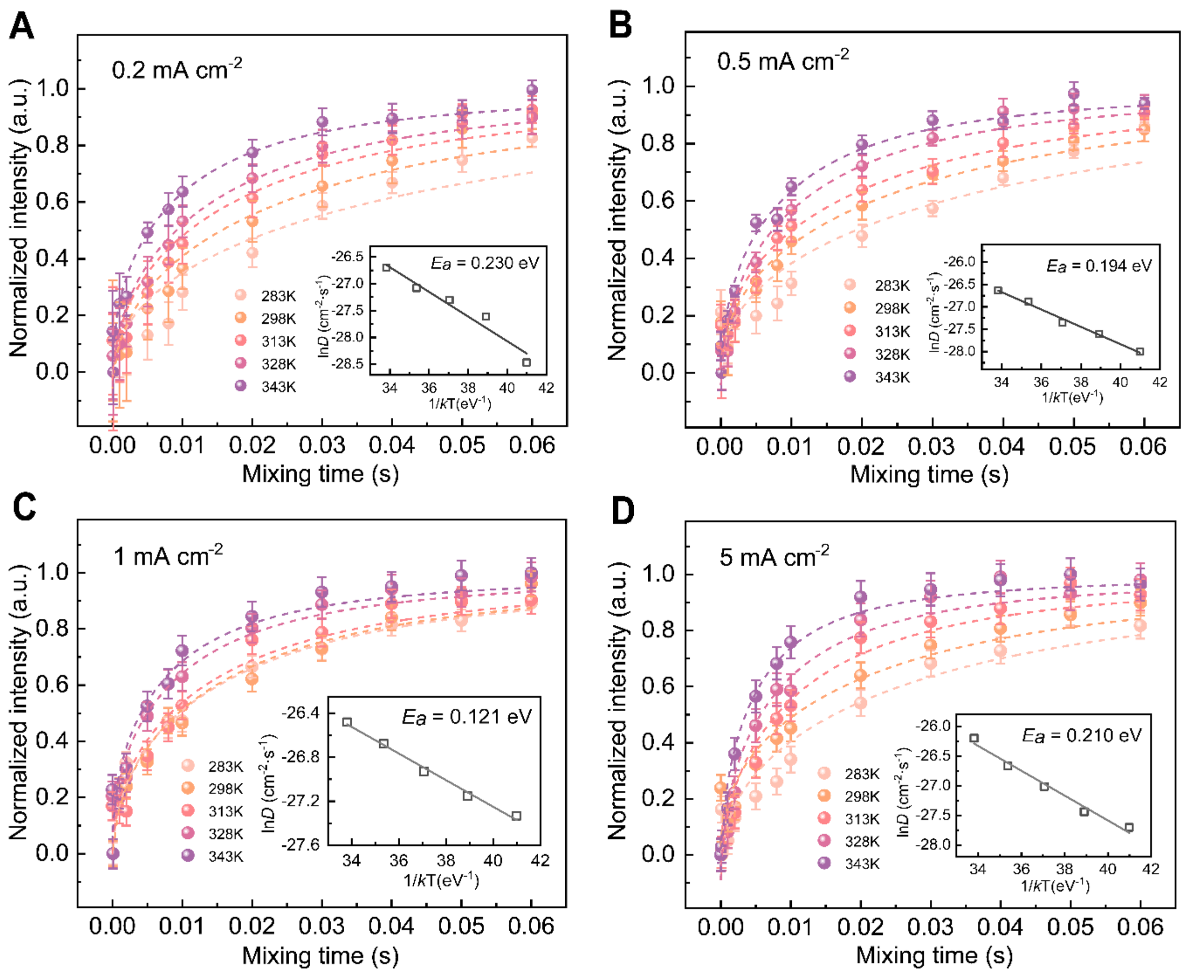


**Fig. S9**. **One-dimensional $^{7}$Li exchange spectroscopy (1D-EXSY) measurements of the ED-Li and the SEI.** (**A**) 0.2 mA cm$^{-2}$, (**B**) 0.5 mA cm$^{-2}$, (**C**) 1 mA cm$^{-2}$ and (**D**) 5 mA cm$^{-2}$. Error bars represent the standard deviation of the spectrum noise.

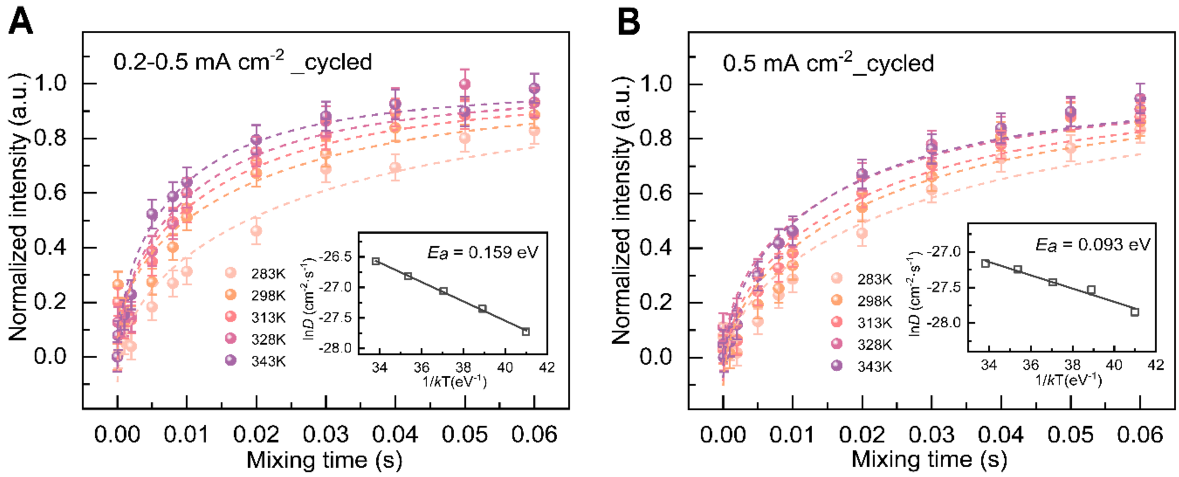


**Fig. S10**. **One-dimensional $^{7}$Li exchange spectroscopy (1D-EXSY) measurements of the ED-Li and the SEI from the cycled samples.** (**A**) 0.2-0.5 mA cm$^{-2}$, (**B**) 0.5 mA cm$^{-2}$. Five formation cycles at 0.2 mA cm$^{-2}$ are first employed, followed by ten cycles at 0.5 mA cm$^{-2}$. One final deposition process at 0.5 mA cm$^{-2}$ is adopted at the end of the cycles to keep the sample amount consistent. Error bars represent the standard deviation of the spectrum noise.

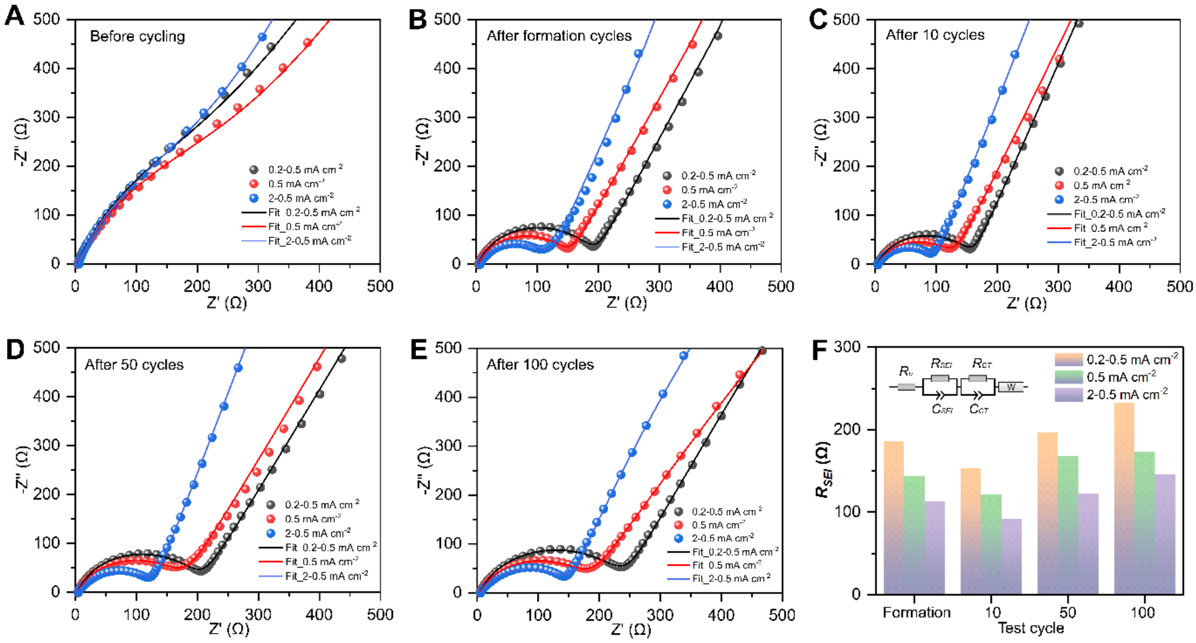


**Fig. S11**. **Electrochemical impedance spectroscopy (EIS) measurements of the cells cycled with different formation current densities.** (**A**) Before cycling and after (**B**) 5 formation cycles, (**C**) 10 working cycles, (**D**) 50 working cycles and (**E**) 100 working cycles. (**F**) comparison of SEI resistance after formation and 10, 50, 100 working cycles. Insert is the equivalent circuit. $R_b$ is the bulk electrolyte resistance, $R_{SEI}$ is the SEI resistance, $R_{ct}$ is the charge-transfer resistance, CPE is constant phase element, the respective CPE describes the capacitance of the corresponding process, and *W* is Warburg diffusion term.

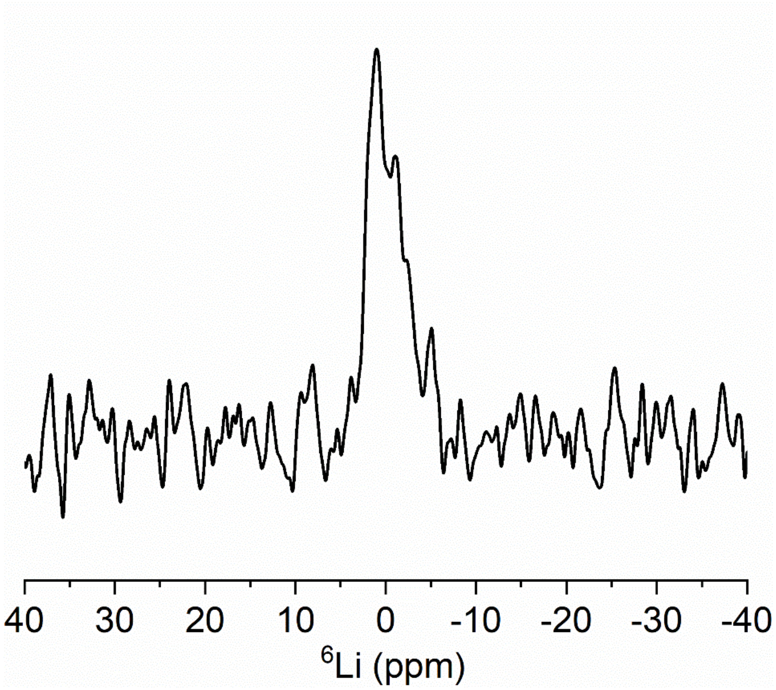


**Fig. S12**. **1D single-pulse $^{6}$Li spectrum of the SEI from the sample plated at 2 mA cm$^{-2}$ (ED-2).**

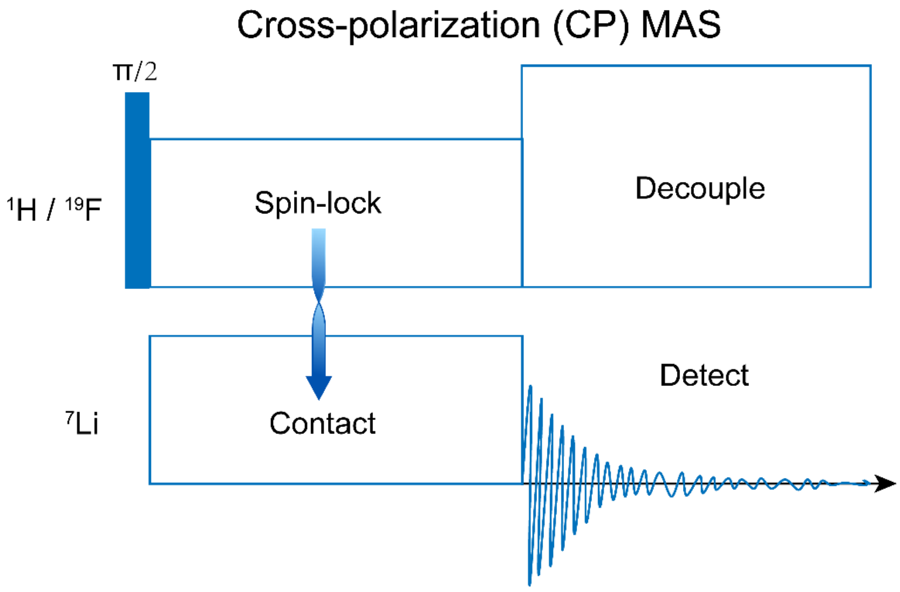


**Fig. S13**. **Schematic showing the pulse sequence employed for the cross-polarization (CP) MAS ssNMR.**

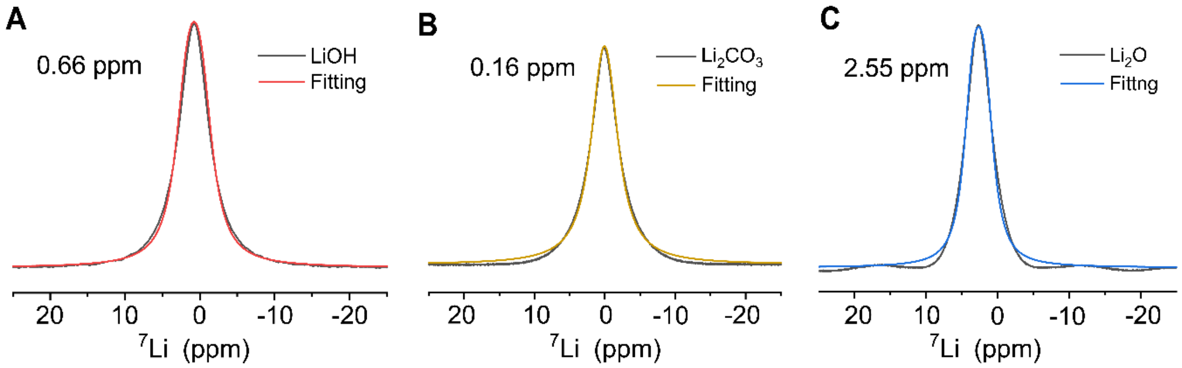


**Fig. S14**. **1D single-pulse $^{7}$Li spectra.** (**A**) LiOH, (**B**) $Li_2CO_3$ and (**C**) $Li_2O$.

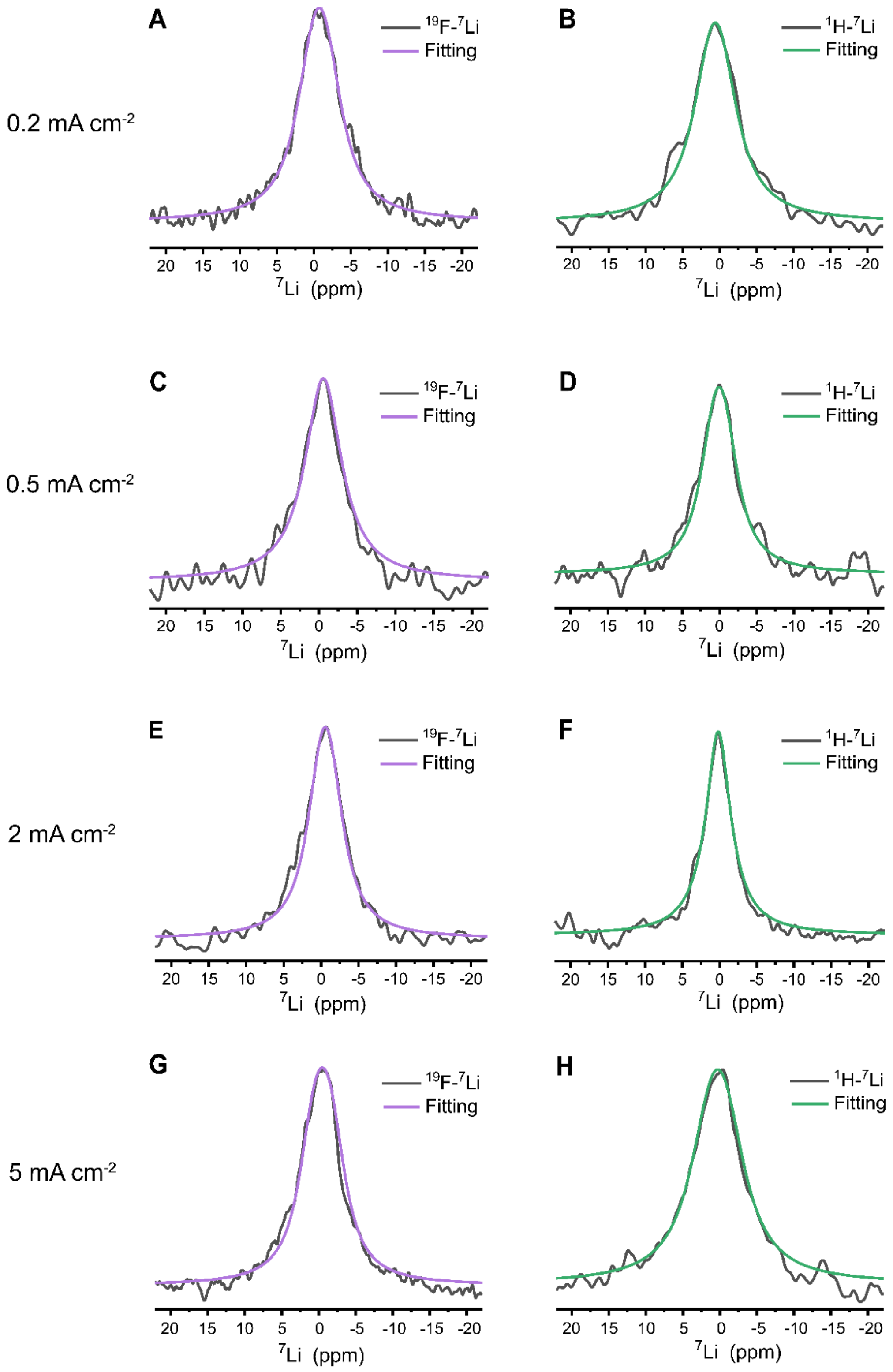


**Fig. S15. Cross-polarization (CP) MAS spectra and the fitting of $^{19}$F→$^{7}$Li, $^{1}$H→$^{7}$Li of the SEI peak plated at different current densities.** (**A** and **B**) 0.2 mA cm$^{-2}$, (**C** and **D**) 0.5 mA cm$^{-2}$, (**E** and **F**) 2 mA cm$^{-2}$ and (**G** and **H**) 5 mA cm$^{-2}$.

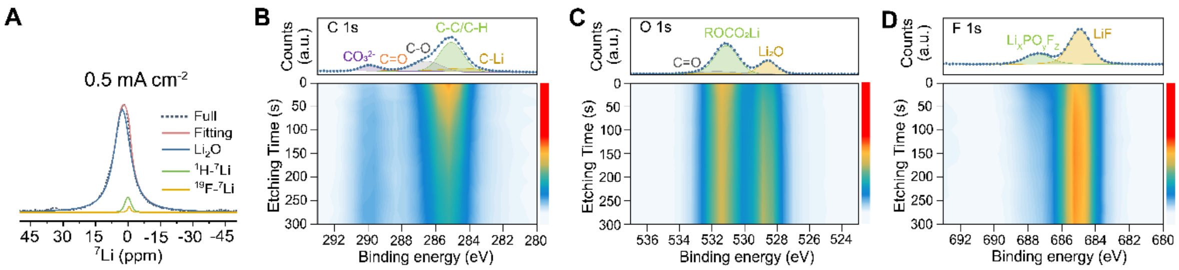


**Fig. S16. Determining the composition of the SEI on ED-Li initially plated at 0.5 mA cm$^{-2}$ using ssNMR and XPS.** (**A**) SEI peak deconvolution for the plating current densities of 0.5 mA cm$^{-2}$ by combining the fitting of the cross-polarization (CP) MAS spectra of $^{1}H \rightarrow ^{7}Li$ (Li near H), $^{19}F \rightarrow ^{7}Li$ (Li near F) and one-pulse measurement of bulk $Li_2O$. Depth-profiled X-ray photoelectron spectroscopy (XPS) measurements of (**B**) C 1s, (**C**) O 1s and (**D**) F 1s for the ED-Li and SEI plated on Cu at 0.5 mA cm$^{-2}$. Color-bar indicates the intensity from weak to strong from bottom to top.

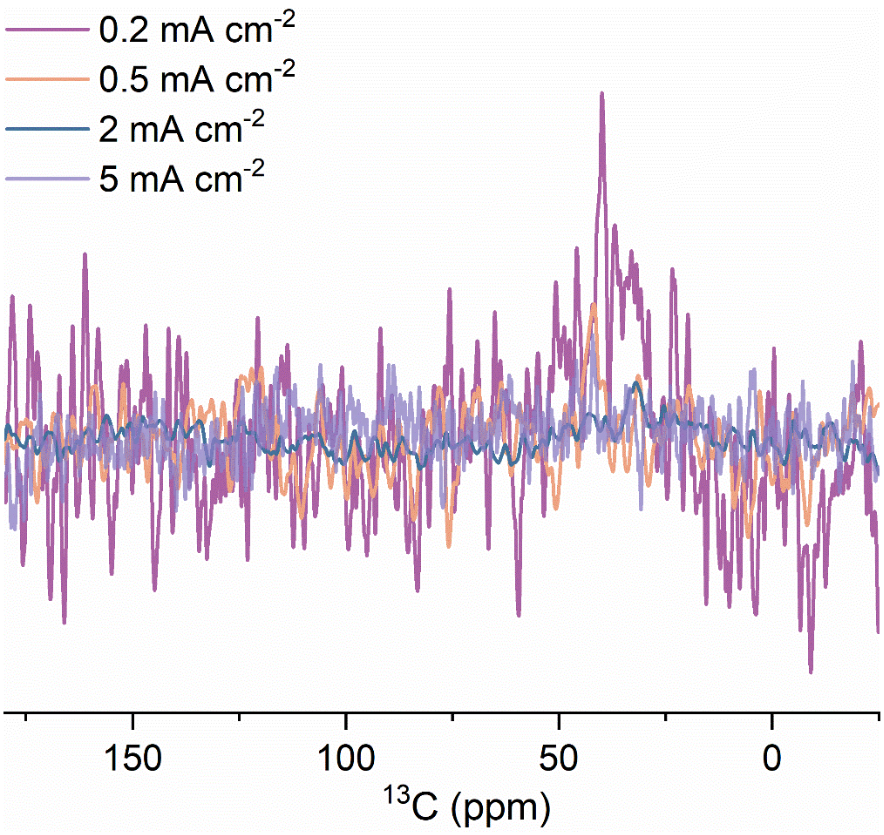


**Fig. S17.** $^{1}H \rightarrow ^{13}C$ **CP MAS NMR spectra of the ED-Li and the corresponding SEI on plating at 0.2 mA cm$^{-2}$, 0.5 mA cm$^{-2}$, 2 mA cm$^{-2}$ and 5 mA cm$^{-2}$, respectively.**

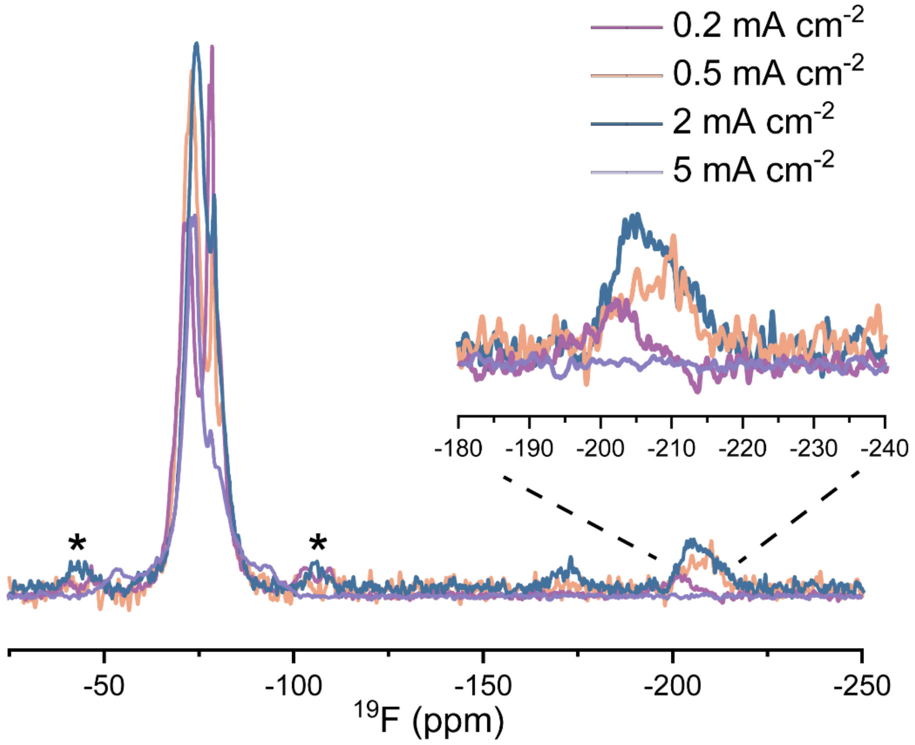


**Fig. S18. 1D single-pulse $^{19}F$ spectra of the ED-Li and the corresponding SEI on plating at 0.2 mA $cm^{-2}$, 0.5 mA $cm^{-2}$, 2 mA $cm^{-2}$ and 5 mA $cm^{-2}$, respectively.** Asterisks denote spinning sidebands.

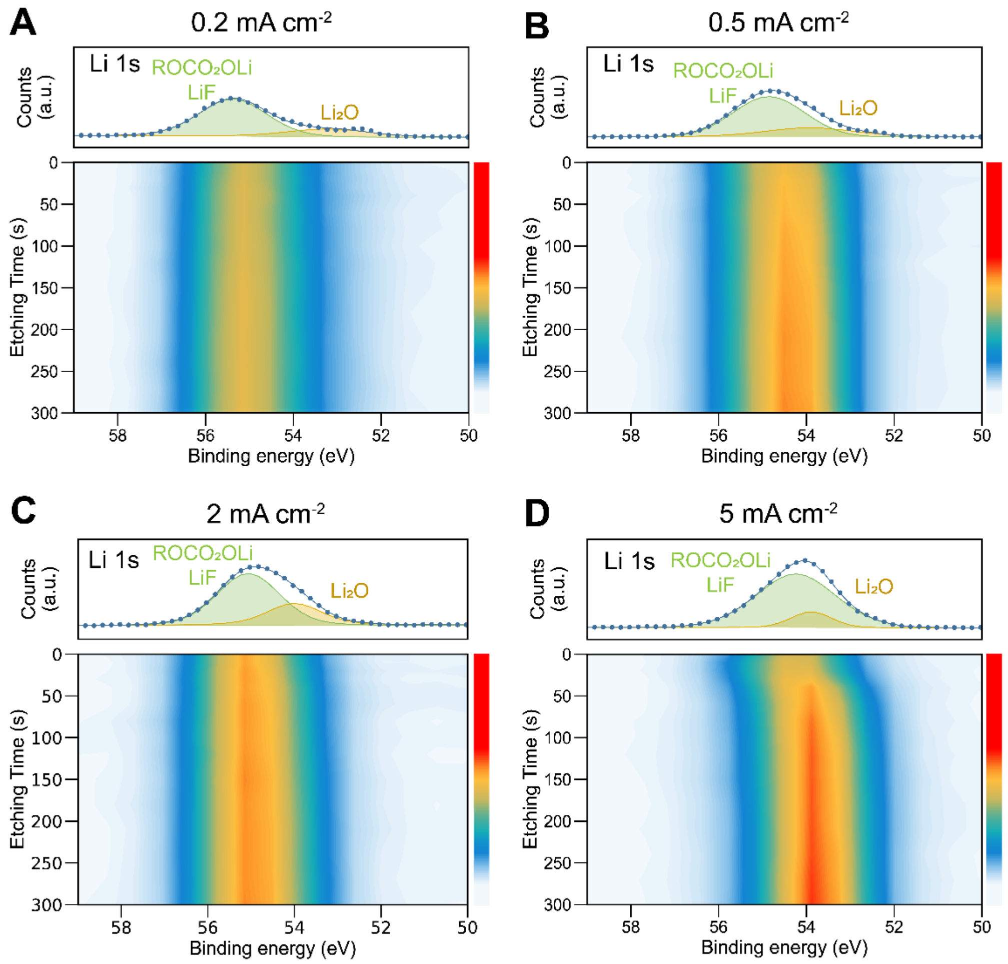


**Fig. S19. Depth-profiled X-ray photoelectron spectroscopy (XPS) measurements of Li 1s for the ED-Li plated on Cu.** (**A**) 0.2 mA $cm^{-2}$, (**B**) 0.5 mA $cm^{-2}$, (**C**) 2 mA $cm^{-2}$ and (**D**) 5 mA $cm^{-2}$. Color-bar indicates the intensity from weak to strong from bottom to top.

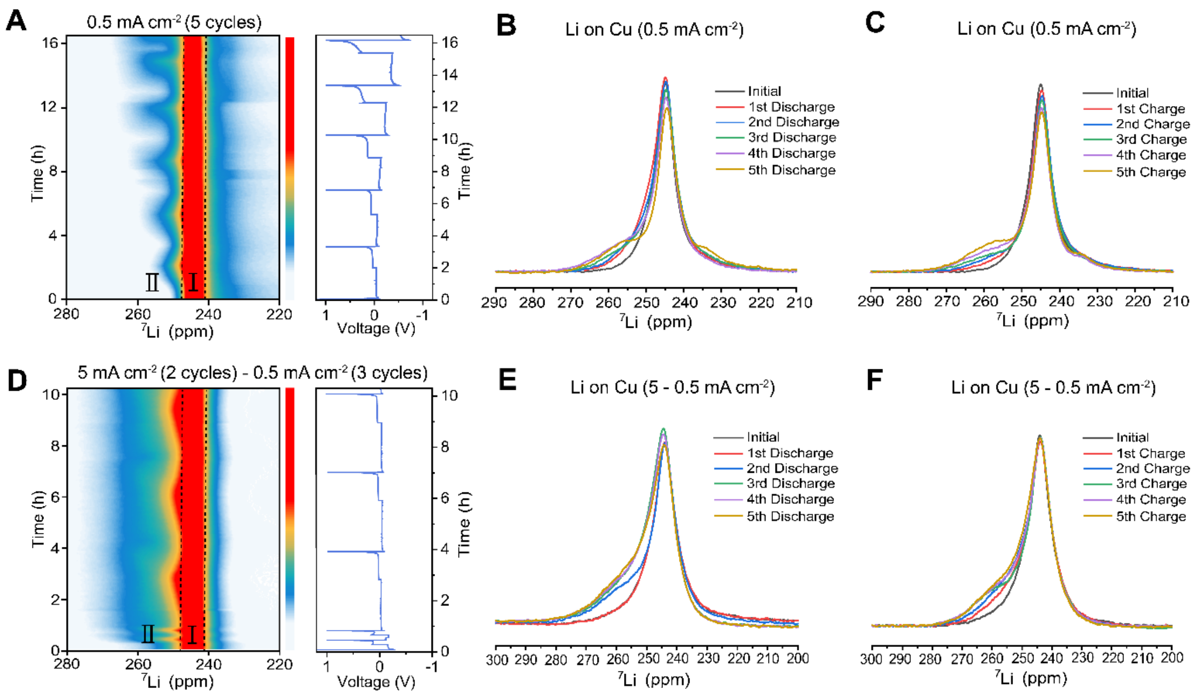


**Fig. S20. Operando NMR study of the evolution of dead Li for the Li||Cu cells cycled with different formation current densities.** Contour plots of the $^{7}Li$ NMR spectra acquired during the plating and stripping of Li metal at (**A**) 0.5 mA cm$^{-2}$ and (**D**) 5-0.5 mA cm$^{-2}$ (pre-cycled at 5 mA cm$^{-2}$ for two cycles and followed by three working cycles at 0.5 mA cm$^{-2}$), along with the electrochemical voltage profile. $^{7}Li$ NMR spectra at the end of (**B** and **E**) discharge and (**C** and **F**) charge for the five cycles, along with the initial Li peak. Color-bar indicates the intensity from weak to strong from bottom to top.

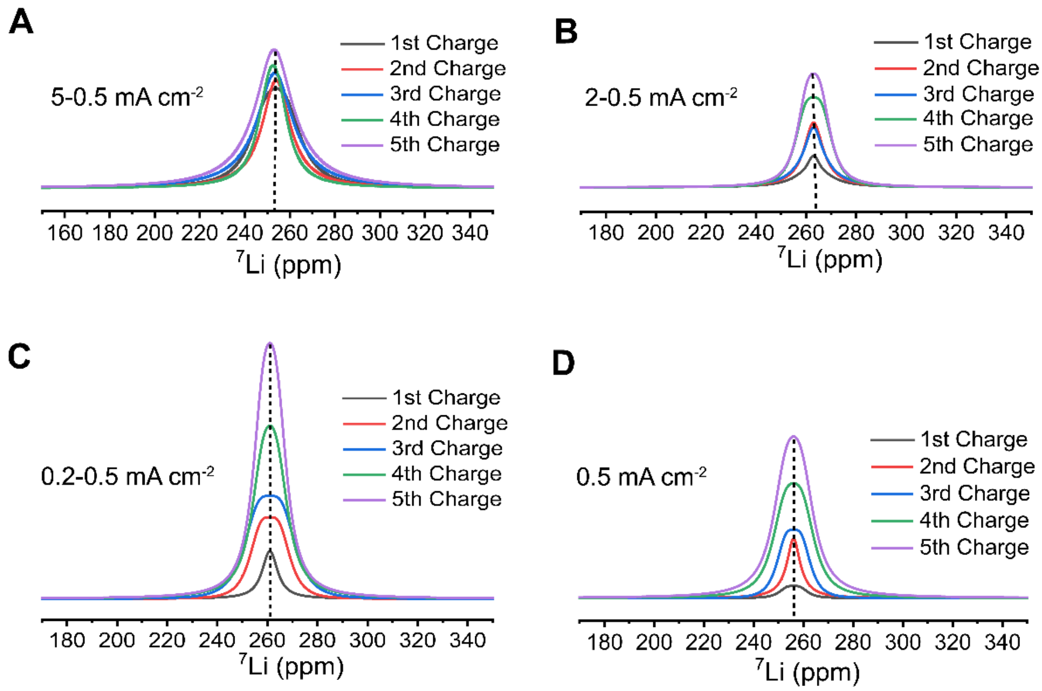


**Fig. S21. $^7$Li NMR signals at the end of charge (stripping) of the operando NMR cells.** (**A**) 5-0.5 mA cm$^{-2}$, (**B**) 2-0.5 mA cm$^{-2}$, (**C**) 0.2-0.5 mA cm$^{-2}$ and (**D**) 0.5 mA cm$^{-2}$. The spectra are extracted from the fitting results of the corresponding $^7$Li NMR slices for the cells cycled in Fig. 5. A and D, and Fig. S20. A and D, respectively.

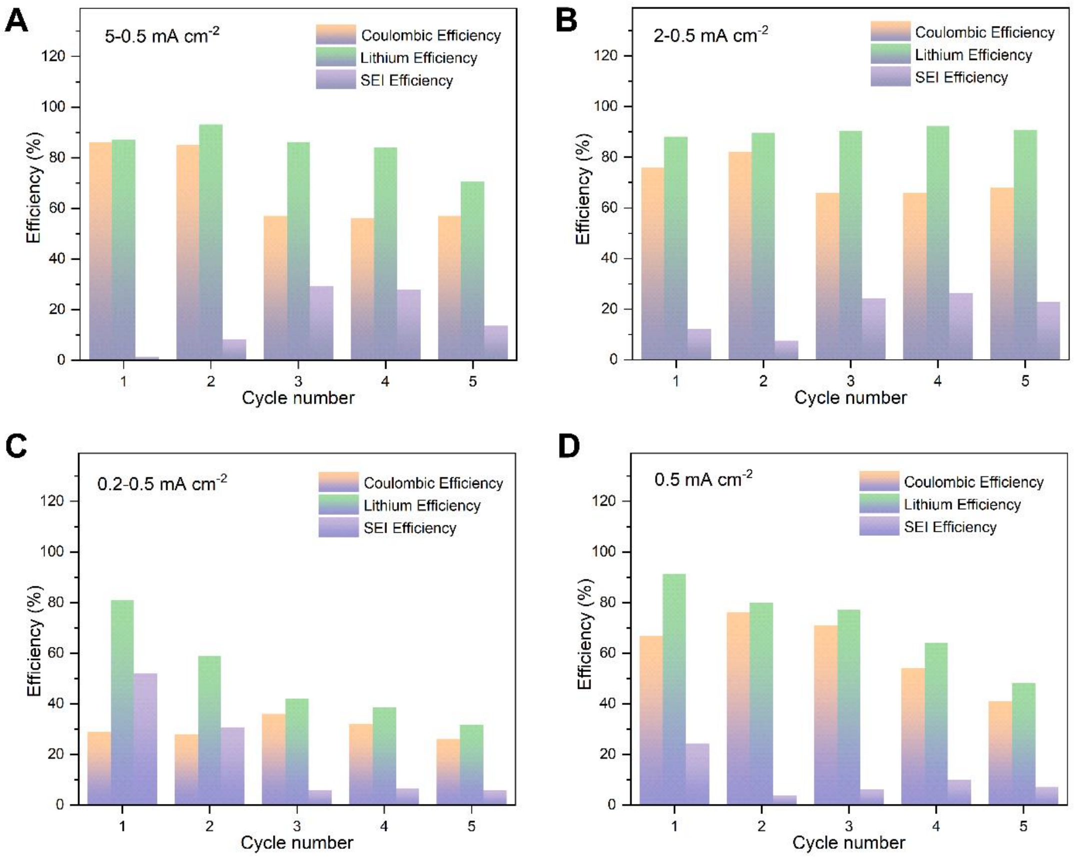


**Fig. S22. Lithium efficiency ($LE_{Li}$) and SEI efficiency ($CE_{SEI}$) obtained from the operando NMR measurements, and Coulombic efficiency (CE) from the electrochemistry of the cells cycled with different formation current dentisies.** (**A**) 5-0.5 mA $cm^{-2}$, (**B**) 2-0.5 mA $cm^{-2}$, (**C**) 0.2-0.5 mA $cm^{-2}$ and (**D**) 0.5 mA $cm^{-2}$.

**Table S1. Average Coulombic efficiency (CE) for the operando NMR cells**

| Cycling protocol ($mA\ cm^{-2}$) | Average CE (×100%) |
|---|---|
| 0.2-0.5 | 0.302 |
| 0.5 | 0.618 |
| 2-0.5 | 0.716 |
| 5-0.5 | 0.682 |
